# Molecular insights into *Candida auris* glycosylphosphatidylinositol transamidase

**DOI:** 10.1093/procel/pwag023

**Published:** 2026-03-23

**Authors:** Xuyang Ding, Zhengkang Hua, Di Zhang, Jiameng Li, Yan Ke, Chu Qi, Ping Yang, Xinlin Hu, Junbo Liu, Mingjie Zhang, Min Zhang, Xiaotian Liu, Hongjun Yu

**Affiliations:** Department of Biochemistry and Molecular Biology, School of Basic Medicine, Tongji Medical College and State Key Laboratory for Diagnosis and Treatment of Severe Zoonotic Infectious Diseases and Hubei Key Laboratory of Natural Active Polysaccharides, Huazhong University of Science and Technology, Wuhan 430030, China; Department of Pathogen Biology, School of Basic Medicine, Tongji Medical College, Huazhong University of Science and Technology, Wuhan 430030, China; Department of Biochemistry and Molecular Biology, School of Basic Medicine, Tongji Medical College and State Key Laboratory for Diagnosis and Treatment of Severe Zoonotic Infectious Diseases and Hubei Key Laboratory of Natural Active Polysaccharides, Huazhong University of Science and Technology, Wuhan 430030, China; Department of Pathogen Biology, School of Basic Medicine, Tongji Medical College, Huazhong University of Science and Technology, Wuhan 430030, China; Department of Pathogen Biology, School of Basic Medicine, Tongji Medical College, Huazhong University of Science and Technology, Wuhan 430030, China; Department of Biochemistry and Molecular Biology, School of Basic Medicine, Tongji Medical College and State Key Laboratory for Diagnosis and Treatment of Severe Zoonotic Infectious Diseases and Hubei Key Laboratory of Natural Active Polysaccharides, Huazhong University of Science and Technology, Wuhan 430030, China; Department of Biochemistry and Molecular Biology, School of Basic Medicine, Tongji Medical College and State Key Laboratory for Diagnosis and Treatment of Severe Zoonotic Infectious Diseases and Hubei Key Laboratory of Natural Active Polysaccharides, Huazhong University of Science and Technology, Wuhan 430030, China; Department of Biochemistry and Molecular Biology, School of Basic Medicine, Tongji Medical College and State Key Laboratory for Diagnosis and Treatment of Severe Zoonotic Infectious Diseases and Hubei Key Laboratory of Natural Active Polysaccharides, Huazhong University of Science and Technology, Wuhan 430030, China; Department of Biochemistry and Molecular Biology, School of Basic Medicine, Tongji Medical College and State Key Laboratory for Diagnosis and Treatment of Severe Zoonotic Infectious Diseases and Hubei Key Laboratory of Natural Active Polysaccharides, Huazhong University of Science and Technology, Wuhan 430030, China; Department of Biochemistry and Molecular Biology, School of Basic Medicine, Tongji Medical College and State Key Laboratory for Diagnosis and Treatment of Severe Zoonotic Infectious Diseases and Hubei Key Laboratory of Natural Active Polysaccharides, Huazhong University of Science and Technology, Wuhan 430030, China; Department of Neuroscience, School of Life Sciences, Southern University of Science and Technology, Shenzhen 518055, China; Department of Neuroscience, School of Life Sciences, Southern University of Science and Technology, Shenzhen 518055, China; Department of Pathogen Biology, School of Basic Medicine, Tongji Medical College, Huazhong University of Science and Technology, Wuhan 430030, China; Department of Neuroscience, School of Life Sciences, Southern University of Science and Technology, Shenzhen 518055, China; Department of Biochemistry and Molecular Biology, School of Basic Medicine, Tongji Medical College and State Key Laboratory for Diagnosis and Treatment of Severe Zoonotic Infectious Diseases and Hubei Key Laboratory of Natural Active Polysaccharides, Huazhong University of Science and Technology, Wuhan 430030, China; Cell Architecture Research Center, Huazhong University of Science and Technology, Wuhan 430030, China

**Dear Editor,** 

*Candida auris* (*C. auris*) poses an escalating global health threat due to its rapid global dissemination and extensive multidrug resistance, underscoring the urgent need for new antifungal strategies (Chowdhary et al., 2023; Eix and Nett, 2025). The glycosylphosphatidylinositol (GPI) biosynthesis pathway is essential for fungal viability and virulence, making it an attractive antifungal target (Ibe and Munro, 2021; Yadav and Khan, 2018). Supporting this premise, several inhibitors (such as Manogepix, M720, and Jawsamycin) have been developed, with Manogepix having progressed to phase III clinical trials (Fu et al., 2020; Mann et al., 2015; Viriyakosol et al., 2019). As the core component of this pathway, GPI transamidase (GPI-T) complex catalyzes ω-site cleavage of precursor proteins and subsequent GPI attachment (Figs. 1A and S1). Although genetic ablation of human GPI-T subunits is non-lethal, subunit deletion in diverse fungal species typically leads to cell death (Komath et al., 2018; Liu et al., 2021). This essentiality highlights the fungal GPI-T complex as a promising antifungal target and underscores the need for thorough structural and functional characterization to elucidate its distinct features from the mammalian counterpart. While recent structural studies have revealed the architecture of human and yeast GPI-T (Hua et al., 2026; Xu et al., 2023), structural knowledge of GPI-T from any pathogenic fungi remains unknown.

**Figure 1.**
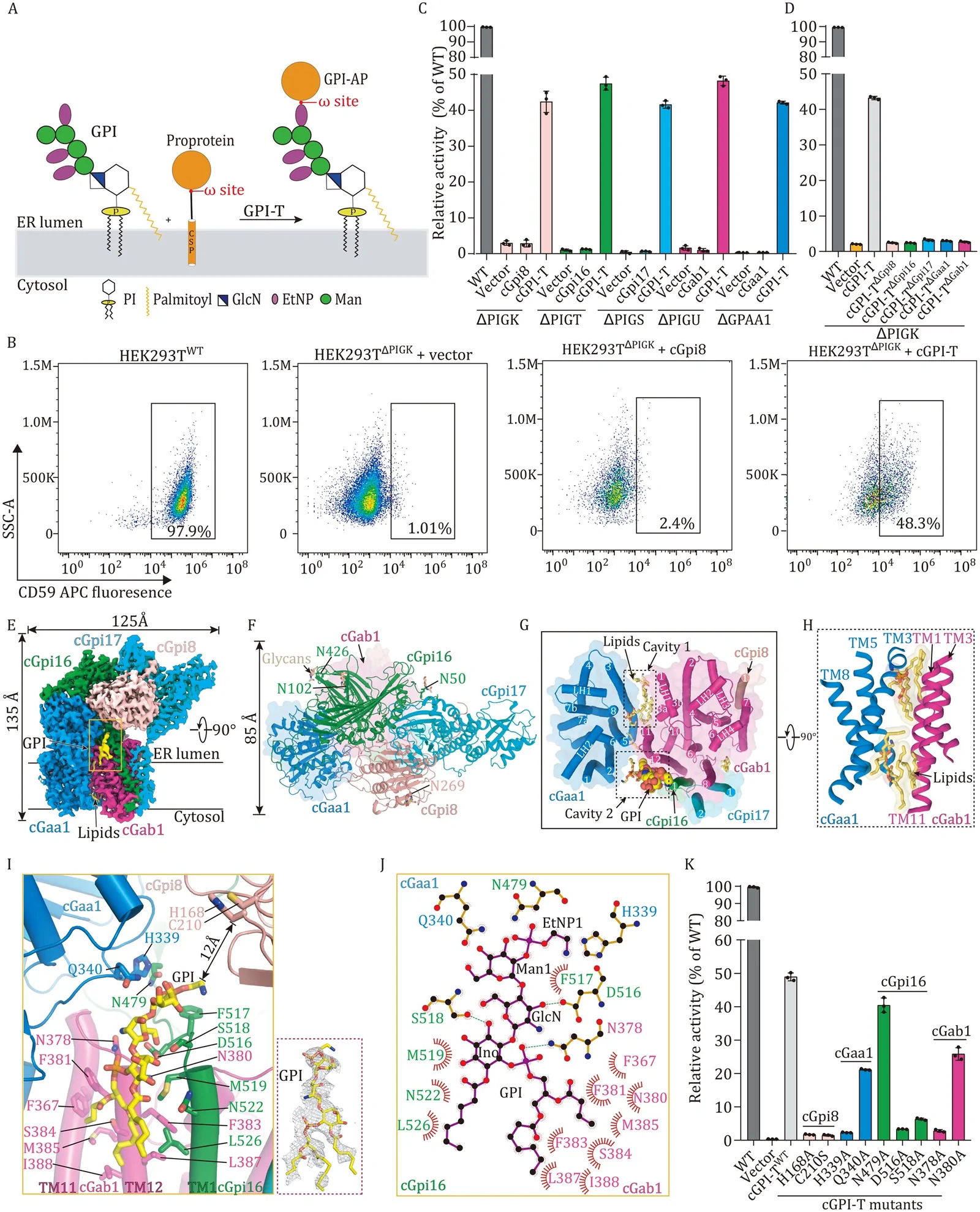
Functional characterization and structure analysis of *C. auris* GPI-T. (A) Schematic of the *C. auris* GPI-T function. GPI-T cleaves the proprotein at the ω-site and links it to ethanolamine phosphate (EtNP) on Man3 of the GPI precursor in the ER lumen. CSP, C-terminal signal peptide; EtNP, ethanolamine phosphate; GlcN, glucosamine; Man, mannose, PI, phosphatidylinositol. (B) *C. auris* GPI-T activity assay. *PIGK*-knockout (KO) HEK293T cells (HEK293T^ΔPIGK^) expressing *C. auris* Gpi8 (cGpi8) or the full GPI-T complex were analyzed using CD59 as a GPI-anchored protein reporter. CD59 surface expression was quantified by FACS using an iF647-conjugated anti-CD59 antibody (detected in the APC channel). Data were analyzed following the gating strategy described in “Materials and Methods” section and Fig. S5. Notably, only co-expression of all five *C. auris* GPI-T subunits markedly restored CD59 surface staining. (C) In HEK293T cells with knockout (KO) of individual GPI-T subunit, either the corresponding *C. auris* GPI-T homolog or the intact GPI-T complex was expressed to assess functional rescue. Quantitative data are shown as the percentage relative to wild-type (WT), based on FACS analysis in (Fig. S2A). The graph represents the mean ± SEM of three independent experiments. None of the individual *C. auris* subunits was sufficient to restore GPI-T activity, whereas co-expression of all five subunits robustly rescued functional activity. (D) In *PIGK*-KO cells, any combination of four of *C. auris* GPI-T or the intact five-subunit GPI-T complex was co-expressed to assess functional rescue. Quantitative data are shown as the percentage relative to WT, based on FACS analysis in (Fig. S2B). The graph represents the mean ± SEM of three independent experiments. None of the four-subunit combinations restored CD59 surface staining, whereas co-expression of all five subunits resulted in a robust recovery. (E) Cryo-EM map of *C. auris* GPI-T complex. Individual subunits are color-coded as indicated. The bound GPI (yellow) and ordered lipids (gray) are labeled. (F) Top view of GPI-T luminal region. The luminal domain is shown in a ribbon representation with N-glycans labelled. The transmembrane domain (TMD) is displayed as transparent surface. (G) Top view of TMD. For clarity, the luminal domain is hidden. TM helices (TMs, denoted by numbers) are depicted as cylindrical helices overlaid with a transparent surface, and the dashed boxes highlight two cavities in the TMD. (H) Close-up view of cavity 1 outlined by the dashed box in (G). TMs mediating TM-lipids interactions are shown in ribbon representation, and interacting lipids are depicted as sticks overlaid with transparent surface. (I) Close-up view of GPI-binding site outlined by the orange box in (E). Key residues of Gab1 (red sticks), Gpi16 (green sticks), and Gaa1 (marine sticks) collectively form a binding pocket that accommodates the GPI ligand (yellow sticks). The catalytic dyad (H168 and C210) of Gpi8 is positioned approximately 12 Å above the bound GPI. The inset (red dashed box) shows the density fitting of modeled GPI. (J) LigPlot view of the interactions between GPI and GPI-T. Hydrophobic interactions are shown as spoked arcs, while hydrogen bonds are depicted as green dashed lines. GPI-T residues follow the same color scheme as in (I). (K) *In vivo* functional analysis of GPI-binding mutations. GPI-T activity was assayed on *PIGK-KO* cells with the introduction of GPI-T WT or mutants. Quantitative data are shown as the percentage relative to WT, based on FACS analysis in (Fig. S2C). The graph represents the mean ± SEM of three independent experiments.

In the absence of a suitable functional assay for *C. auris* GPI-T, we developed a flow cytometry (FACS)-based assay using CD59 as a GPI-anchored reporter in HEK293T knockout (KO) cells. Notably, deletion of human *PIGK* (the homolog of *C. auris* Gpi8, cGpi8) led to a severe loss of CD59 surface expression. Sole expression of cGpi8 failed to rescue this phenotype, whereas co‑expression of the complete five‑subunit *C. auris* GPI‑T complex robustly restored CD59 surface levels (Fig. 1B). To extend this finding, we generated KO cell lines for each of the four remaining human GPI-T subunits (hPIGT, hPIGS, hGPAA1, and hPIGU) and attempted rescue with either individual *C. auris* homologs (cGpi16, cGpi17, cGaa1, and cGab1) or the intact five-subunit *C. auris* GPI-T complex. Consistent with the above findings, individual *C. auris* subunits did not restore CD59 surface expression in the corresponding KO cells, whereas only the full *C. auris* complex strongly rescued surface localization (Figs. 1C and S2A). Moreover, we also evaluated partial complexes by transfecting *PIGK*-KO cells with various combinations of four *C. auris* GPI-T subunits. None of these combinations restored CD59 surface expression and functional rescue was achieved only upon the inclusion of all five GPI-T subunits (Figs. 1D and S2B), in line with the results observed with other GPI-T subunits (Fig. 1C). Collectively, these results suggest substantial interspecies divergence, as functional assembly of *C. auris* GPI-T strictly requires all five fungal subunits and cannot be complemented by their human counterpart.

To further uncover the molecular architecture of *C. auris* GPI-T, we reconstituted the fully assembled *C. auris* GPI-T complex and determined its cryo-EM structure at 3.2 Å resolution (Fig. S3). The resulting cryo-EM map reveals a pentameric assembly comprising subunits Gpi8, Gpi16, Gaa1, Gpi17, and Gab1 in a 1:1:1:1:1 stoichiometry (Fig. 1E). The *C. auris* GPI-T complex measures approximately 125 × 85 × 135 Å and comprises a luminal region and a transmembrane domain (TMD) (Fig. 1E–G). The luminal region is formed by the soluble domains of Gpi8, Gpi16, Gpi17, and Gaa1 (Fig. 1F). Specifically, Gpi16 and Gpi8 constitute a central structural scaffold flanked by Gaa1 and Gpi17 from both sides, collectively stabilizing the GPI-T luminal region (Fig. 1F). Within this region, four N-glycosylation sites were identified: three on Gpi16 and one on Gpi8 (Figs. 1F and S4). Among these, only N102 on Gpi16 is highly conserved across diverse fungal species (Fig. S9). The TMD region comprises 24 transmembrane helices (TMs) from 5 subunits (Fig. 1G). Specifically, TMs of Gpi17, Gpi16, Gpi8, and Gab1 form a discrete structural ensemble, which associates loosely with the TMs of Gaa1 through hydrophobic contacts between Gab1 TM11 and Gaa1 TM5 (Fig. 1G). The organization creates two distinct membrane-embedded cavities for lipid accommodation (Fig. 1G). Cavity 1, formed by Gab1 (TM1, TM3, and TM11) and Gaa1 (TM3, TM5, and TM8), harbors nine tightly packed lipid molecules that stabilize the TMD (Figs. 1H and S4). Cavity 2, as a wider and flatter pocket shaped by Gab1 (TM11 and TM12), Gpi16 (TM1), and Gaa1 (TM1, TM2, and TM5), is identified as the binding site for GPI (Fig. 1G).

Within the cavity 2, we observed an additional lipid-like density that extends into the luminal space between the soluble domains of Gaa1, Gpi16, and Gpi8, reaching within 12 Å of the catalytic residues (H168 and C210) of Gpi8 (Fig. 1I) (Meyer et al., 2000; Sah et al., 2020). Accordingly, we modeled a GPI molecule consisting of an alkyl-phosphatidylinositol (acylPI), a glucosamine (GlcN), a mannose (Man1), and an ethanolamine phosphate (EtNP1) (Fig. 1I and 1J, inset). Notably, residues lining the GPI-binding site are highly conserved (Fig. S8), supporting its role as the functional core of the GPI-T complex. The GPI engages in extensive interactions with multiple subunits. Specifically, its hydrophilic moiety forms polar contacts with N479, D516 and S518 of Gpi16, N378 of Gab1, H339 and Q340 of Gaa1 as well as hydrophobic interaction with Gpi16 F517 (Fig. 1I and 1J). Meanwhile, the three alkyl chains of GPI insert into the transmembrane region of GPI-T, engaging in van der Waals interactions with M519, N522, and L526 of Gpi16, as well as with F367, N380, F381, F383, S384, M385, L387, and I388 of Gab1. Consistent with our structural observations, mutations H339A (Gaa1), D516A and S518A (Gpi16), and N378A (Gab1) each severely impaired GPI-T activity (by >90%), akin to catalytically inactive Gpi8 mutants (H168A and C210S) (Figs. 1K and S2C). Activity reductions of >50% were observed for Q340A (Gaa1) and N380A (Gab1), while N479A (Gpi16) is the only mutant with mild effects (Figs. 1K and S2C). These results integrate structural and functional evidence, establishing the conserved GPI-binding pocket as essential for GPI-T activity.

Importantly, our determined *C. auris* GPI-T structure uncovered considerable divergence from its human counterpart (Fig. 2A–H), which could facilitate the development of strategies to specifically target *C. auris* GPI-T. The most pronounced structural differences are concentrated at the interaction interfaces surrounding Gpi8, the key catalytic subunit (Benghezal et al., 1996; Meyer et al., 2000). At the Gpi8–Gaa1 interface, we identified a Gpi8 loop that corresponds to an auto-inhibitory segment in human GPI-T (Xu et al., 2023) (Figs. 2C and S6A). This segment in humans occupies the active site in the closed state and repositions upon substrate binding. Intriguingly, in *C. auris* GPI-T, this loop adopts a substrate-bound open conformation even without a protein substrate present. Sequence alignment indicates this loop is highly conserved among pathogenic fungi but diverges from humans (Fig. 2D), suggesting distinct catalytic activation mechanisms between fungal and human GPI-T systems. At the Gpi8–Gpi17 interface, a C-terminal anchoring loop of Gpi8 (residues 312–326) follows a substantially altered path compared to the corresponding region in human PIGK (Fig. 2E). This structural deviation is accommodated by conformational rearrangements in several adjacent loops of *C. auris* Gpi17, including Lα5–α6, Lβ10–β11, and Lβ3–β4 (Figs. 2E and S6B–D). Following the C-terminal anchoring loop, a large fragment (residues 327–365) of Gpi8 in the *C. auris* structure was poorly resolved due to conformational flexibility. In contrast, the equivalent region in human GPI-T is well-ordered and mediates critical interactions with hPIGT (the human homolog of Gpi16) (Fig. 2F). In the human complex, three loops of hPIGT (corresponding to Lα1–β5, Lβ6–β7, and Lβ22–β23) converge to form a stable binding platform for hPIGK. Strikingly, this platform is disrupted in the *C. auris* complex, where the equivalent loops are shortened and displaced outward, leading to increased flexibility in the corresponding Gpi8 region (Figs. 2F and S6F–H). Notable structural divergences are also present at the Gpi16 interaction interfaces. In *C. auris*, the Lβ2–β3 loop of Gpi16 is elongated by 10 residues compared to human PIGT, extending further toward Gpi17 and enhancing their interfacial stability (Figs. 2G and S6E). Similarly, at the Gpi16–Gaa1–Gpi8 interface, the Lβ7–β8 loop of Gpi16 is extended by 11 residues, forming a clamp-like structure (Figs. 2H and S6G). Its proximal portion contacts Gaa1, while the distal segment engages Gpi8, collectively reinforcing the stability of this trimeric interface (Fig. 2H). Additionally, we extended the structural and sequence comparisons of GPI–T from *C. auris*, *S. cerevisiae*, and humans. Our structural analysis further indicates that interspecies differences are mainly concentrated at subunit-subunit interfaces (Fig. S7A). Among these, several regions are conserved between *C. auris* and *S. cerevisiae* but diverge from humans, including the Lβ7–β8 and Lβ22–β23 loops at the Gpi16–Gaa1–Gpi8 and Gpi8–Gpi16 interfaces, respectively (Fig. S7B–C and S7I–J). Though the auto-inhibitory loop at the Gpi8–Gaa1 interface is not resolved in *S. cerevisiae*, its sequence is conserved between the two fungi but diverges from humans (Fig. S7D). In contrast, regions divergent in three species include the Lα1–β5 and Lβ6–β7 loops at the Gpi8–Gpi16 interface; the Lβ2–β3 loop at the Gpi16–Gpi17 interface; and the Lβ3–β4, Lβ10–β11, and Lα5–α6 loops at the Gpi8–Gpi17 interface (Fig. S7C, S7E–I, and S7K–M).

**Figure 2.**
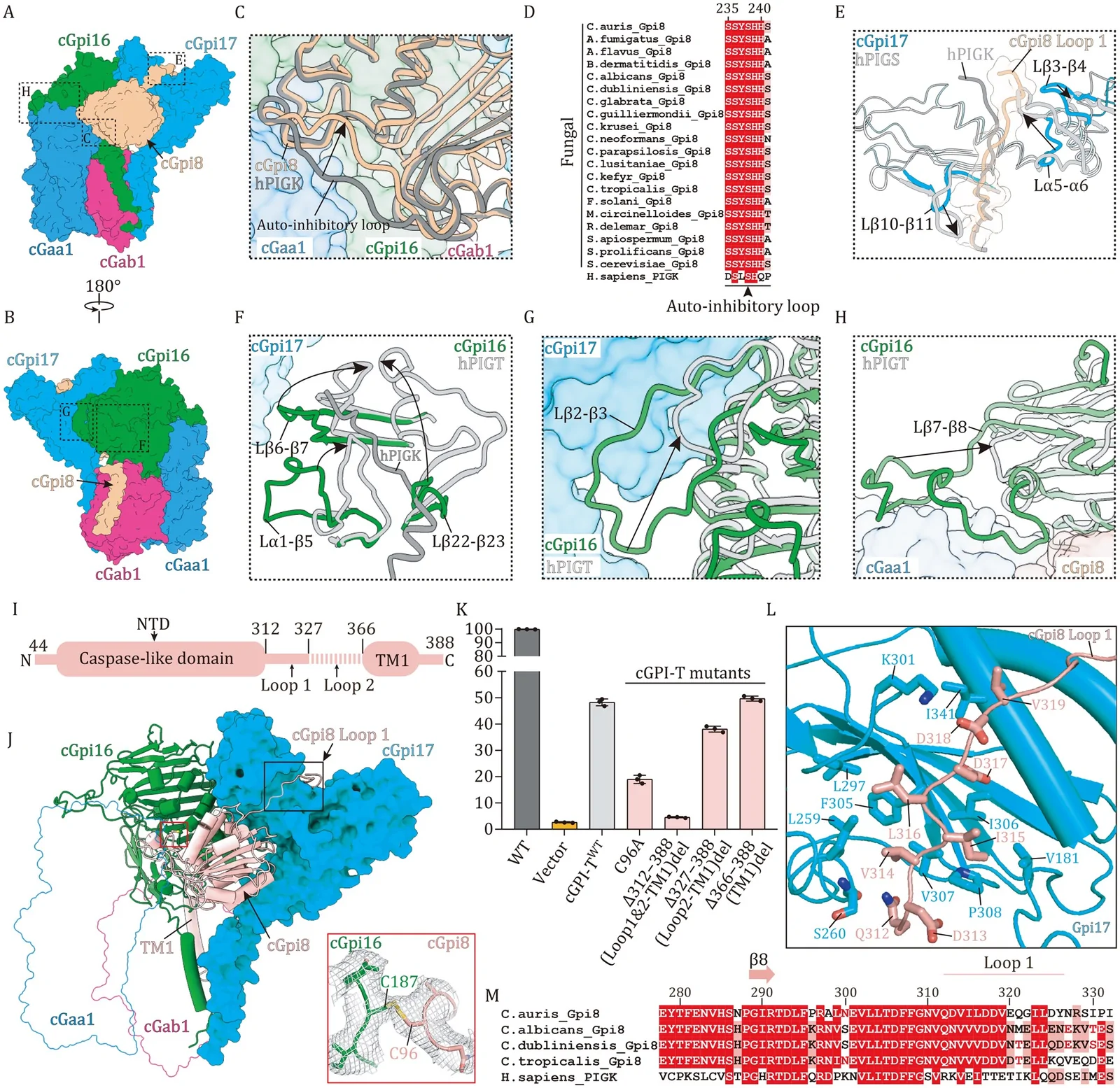
Distinct structural features of GPI-T in *C. auris* relative to human. (A and B) Schematic views of regions displaying notable structural differences between *C. auris* and human GPI-T (PDB ID: 7WLD). For clarity, only the *C. auris* GPI-T complex is shown in front view (A) and back view (B). The identified structural differences are detailed in panels (C–H). (C and D) Structural comparisons (C) and sequence alignments (D) in the auto-inhibitory loop of Gpi8 between *C. auris* and human. Major structural difference is indicated by an arrow. (E–H) Close-up view of the structural differences at multiple subunit interfaces: Gpi8–Gpi17 (E), Gpi8–Gpi16 (F), Gpi16–Gpi17 (G), and Gpi16-Gaa1-Gpi8 (H). Major structural differences are indicated by arrows, and the corresponding structural elements are annotated in the sequence alignments (Fig. S6B–H). (I) Schematic representation of Gpi8 showing the N-terminal domain (NTD), two loop regions (Loop1 and Loop2), and a single TM helix (TM1). Loop2 is depicted as dashed lines to indicate its intrinsic flexibility. (J) The binding mode of Gpi8. Gpi8 (salmon) and Gpi16 (green) are shown in ribbon representation, Gpi17 (cyan) is shown as a surface, and Gaa1 (blue) and Gab1 (red) are depicted in wire representation. The inset shows the disulfide bond between Cys187 of Gpi16 and Cys96 of Gpi8 as marked by red box in (J). (K) *In vivo* functional analysis of Gpi8 mutations. GPI-T activity was assayed on *PIGK-KO* cells with the introduction of GPI-T WT or mutants. Quantitative data are shown as the percentage relative to WT, based on FACS analysis in (Fig. S2D). The graph represents the mean ± SEM of three independent experiments. (L) Close-up view of the interactions between the Loop1 of Gpi8 and Gpi17, corresponding to the region highlighted by the black box in (J). (M) Multiple sequence alignment of Gpi8 Loop1 across human and major *Candida* pathogens (*C. auris*, *C. albicans*, *C. dubliniensis*, *C. tropicalis*).

Given that Gpi8 serves as the key catalytic subunit of the GPI-T complex, we further investigated several determinants critical for its anchoring. The N-terminal domain (NTD) of Gpi8 adopts a caspase-like fold (Fig. 2I) and is embedded within a cavity formed by Gpi16, Gpi17, and Gaa1 (Fig. 2J). Notably, a disulfide bond between Cys96 of Gpi8 and Cys187 of Gpi16 stabilizes the association of Gpi8 with Gpi16 (Fig. 2J, inset). Consistently, the C96A mutation led to a marked decrease in GPI-T activity (Figs. 2K and S2D). Following the NTD, Gpi8 contains an extended C-terminal region comprising three elements: Loop1 (residues 312–326), Loop2 (residues 327–365), and TM1 (residues 366–386) (Fig. 2I). Specifically, Loop1 spans the central junction of the claw-like architecture formed by Gpi17 luminal domain (Fig. 2J). Loop2 is flexible and was not resolved in the map, while TM1 inserts into the membrane and packs against Gab1 TM7 (Figs. 1G, 2I, and 2J). To assess the functional relevance, we generated a series of C-terminal truncations of Gpi8. The results showed that Loop1 is essential for GPI-T activity, whereas Loop2 and TM1 have only moderate effects (Figs. 2K and S2D). Detailed analysis of the functionally critical Loop1 reveals its extensive interactions with Gpi17. These include polar interactions between Gpi8 residues Q312 and D318 with Gpi17 residues S260 and K301, respectively, as well as hydrophobic contacts involving Gpi8 residues V314, I315, L316, and V319 with Gpi17 residues L259/F305/V307, P308/V181/I306, L297, and I341, respectively (Fig. 2L). Importantly, sequence analysis indicates that Loop1 is conserved among major *Candida* pathogens but shows low conservation in the human homolog (Fig. 2M), suggesting its potential for pathogen-specific therapeutic intervention.

In summary, our structural and mechanistic analyses of the *C. auris* GPI-T complex illuminate its assembly, substrate recognition, and catalytically relevant features, while defining key species-specific divergences from the human enzyme. These insights establish a structural framework for future mechanistic investigations and provide a foundation for the rational development of pathogen-selective antifungal strategies targeting the GPI-T machinery in *C. auris*.

## Supplementary data

Supplementary data is available at *Protein & Cell* Journal online https://doi.org/10.1093/procel/pwag023.

## Footnotes

We thank the staff members of the Cryo-EM Center, Southern University of Science and Technology for assistance in data collection. We thank Prof Yishi Liu of the Glycobiology Laboratory at Jiangnan University for kindly providing the *PIGK*-KO, *PIGS*-KO, *PIGU*-KO, and *PIGT*-KO cell lines used in this study.

This work was supported by the National Natural Science Foundation of China (92478121 to H.Y.), Shenzhen Medical Research Fund (A2303054 to X.L.), the Natural Science Fund for Distinguished Young Scholars of Hubei Province (2024AFA044 to M.Z.), Independent project of State Key Laboratory for Diagnosis and Treatment of Severe Zoonotic Infectious Disease (2025ZZ10002 to M.Z.), National Natural Science Foundation of China (92053112, 31971148 to H.Y., 32100575 to M.Z., 0204K0010 to X.H.), Key-Area Research and Development Program of Guangdong Province (2023B0303010001 to M.Z.), the Fundamental Research Funds for the Central Universities (5003510141, 5003510056 to H.Y. and 5003510112 to M.Z.) and Postdoctoral Fellowship Program of China Postdoctoral Science Foundation (GZB20240245 to X.H.)

The authors declare no competing interests.

The EM density map and atomic coordinates of *Candida auris* GPI-T complex have been deposited and are available in the EMDB under accession codes EMDB-66656 and in the PDB under accession codes 9X8O.

M.Z. and H.Y. conceived the project. M.Z., M.J.Z., X.L., and H.Y. designed experiments. X.D., Z.H., M.Z., and X.L. performed most of the experiments. D.Z., M.J.L., Y.K., C.Q., X.H., P.Y., and B.J.L. helped with the sample preparation and functional experiments. X.D., Z.H., M.J.Z, M.Z., X.L., and H.Y. analyzed the data. M.Z. and H.Y. wrote the manuscript with the help of all the authors.

## Supplementary Material

pwag023_Supplementary_Data
