# Supplementary material for: Molecular insights into Candida auris glycosylphosphatidylinositol transamidase
Source: Protein Cell. 2026 Mar 23;17(10):975–80. doi: 10.1093/procel/pwag023 (PMC13626736; doi:10.1093/procel/pwag023)
Supplement: Supplementary file 1 [file pwag023_supplementary_data.zip › Supplementary_Materials-updated.pdf]

Supporting Information for

Molecular insights into *Candida auris* glycosylphosphatidylinositol transamidase

Xuyang Ding[1,2]*, Zhengkang Hua[1,2]*, Di Zhang[2], Jiameng Li[1], Yan Ke[1], Chu Qi[1], Ping Yang[1], Xinlin Hu[1], Junbo Liu[3], Mingjie Zhang[3], Min Zhang[2#], Xiaotian Liu[3#], Hongjun Yu[1,4#]

[1] Department of Biochemistry and Molecular Biology, School of Basic Medicine, Tongji Medical College and State Key Laboratory for Diagnosis and Treatment of Severe Zoonotic Infectious Diseases and Hubei Key Laboratory of Natural Active Polysaccharides, Huazhong University of Science and Technology, Wuhan 430030, China.
[2]Department of Pathogen Biology, School of Basic Medicine, Tongji Medical College, Huazhong University of Science and Technology, Wuhan 430030, China.
[3]Department of Neuroscience, School of Life Sciences, Southern University of Science and Technology, Shenzhen 518055, China
[4]Cell Architecture Research Center, Huazhong University of Science and Technology, Wuhan 430030, China.

*These authors contributed equally to this work.

#**Corresponding author**: minzhang@hust.edu.cn (M.Z.) or liuxt@sustech.edu.cn (X.L.) or hongjun_yu@hust.edu.cn (H.Y.)

This PDF file includes:

Materials and Methods
Figures S1 to S10
Table S1

Materials and Methods

Plasmids construction

The full-length genes encoding the *C. auris* GPI-T subunits Gaa1 (UniProt ID: A0AAW0VIZ5), Gab1 (A0AAW0VNG9), Gpi8 (A0A0L0P4W1), Gpi16 (A0A2H0ZIP2), and Gpi17 (A0AAW0VID9) were codon-optimized and individually subcloned into the pCAG vector. To facilitate protein identification and FACS, each subunit was fused at its C terminus with a thermostable green fluorescent protein (TGP) tag (Cai et al., 2020; Close et al., 2015). In addition, a Flag tag was appended to the C-terminus of Gpi8 for affinity purification. Site-directed mutagenesis was performed on the parental plasmid to generate the indicated variants. All DNA constructs were confirmed by Sanger sequencing.

C. auris GPI-T expression and purification

The five subunits of *C. auris* GPI-T were co-expressed in human embryonic kidney (HEK) 293F cells (Invitrogen), as detailed below. HEK293F cells were cultured at 37 °C with 5% CO_2 in a shaking incubator (120 r.p.m.) and transfected at a density of 3×10^6 cells/mL. Plasmids encoding the five *C. auris* GPI-T subunits were co-transfected at an equimolar ratio, using 2 mg of total DNA per liter of culture and a polyethyleneimine (PEI) to DNA ratio of 3:1 (w/w). After 48 h, cells were harvested by centrifugation at 4000 × g for 5 minutes at room temperature.

The cell pellet was resuspended in lysis buffer (25 mM Tris-HCl, 150 mM NaCl, pH 7.5) supplemented with 1% (v/v) protease inhibitor cocktail (Roche) and then lysed by sonication. The membrane was solubilized with 1% (w/v) n-dodecyl-β-D-maltopyranoside (DDM, Anatrace) and 0.1% (w/v) cholesteryl hemisuccinate Tris salt (CHS, Anatrace) at 4 °C for 2 hours. After removal of cellular debris by centrifugation at 15,000 × g for 40 minutes, the supernatant was incubated with 1 mL of pre-equilibrated anti-FLAG M2 affinity resin (Sigma) for 2 hours. The resin was then washed with 5 column volumes of buffer A (25 mM Tris-HCl, 150 mM NaCl, pH 7.5, 0.03% glyco-diosgenin (GDN, Anatrace)), followed by eluted with 5 mL of buffer B (25 mM Tris-HCl, 150 mM NaCl, pH 7.5, 0.03% GDN, 0.15 mg/mL 3 × FLAG peptide (GenScript)).

The eluate was further concentrated using a 100-kDa cut-off concentrator (Millipore) and subsequently subjected to SEC (Superose 6 Increase 10/300 GL, GE Healthcare) equilibrated in buffer A. Peak fractions containing the GPI-T complex were identified by both Coomassie blue staining and in-gel fluorescence analysis after SDS-PAGE. The confirmed fractions were pooled and concentrated to ~5 mg/mL for cryo-EM grid preparation.

Generation of human *GPAA1* knockout (KO) cell line

To disrupt human *GPAA1*, CRISPR–Cas9 genome editing was performed in HEK293T cells. A pair of oligonucleotides (GPAA1 KO1: forward 5′-CACCGCTACACGCAGA GTTTCTCC-3′, reverse 5′-AAACGGAGAAACTCTGCGTGTAGC-3′) was cloned into the lentiCRISPRv2 vector to generate a lentiCRISPRv2-sgRNA construct (Shalem et al., 2014), which was confirmed by Sanger sequencing.

Lentiviral particles were produced in HEK293T cells cultured in Dulbecco's Modified Eagle Medium (DMEM, Cytiva) supplemented with 10% fetal bovine serum (FBS, Cegrogen Biotech) inside a stationary incubator (37 °C, 5% CO_2). Cells were seeded in 10-cm dishes one day before transfection to reach 70–80% confluence. For each dish, 4 μg lentiCRISPRv2-sgRNA, 3 μg psPAX2, and 1 μg pMD2.G were mixed in 500 μL Opti-MEM (Gibco, Thermo Fisher Scientific) (Sanjana et al., 2014; Shalem et al., 2014; Wang et al., 2014). Separately, 24 μg polyethyleneimine (PEI) was diluted in 500 μL Opti-MEM. After 5 min incubation, PEI and plasmid solutions were combined, incubated for 15 min at room temperature, and added dropwise to the cells. Culture supernatants were collected at 48 h and 72 h post-transfection, centrifuged at 500 × g for 5 min, and filtered through 0.45 μm polyethersulfone (PES) membranes. For concentration, viral supernatants were ultracentrifuged at 100,000 × g for 2 h at 4 °C. Viral titers were determined by serial dilution infection of HEK293T cells followed by puromycin selection (1μg/mL for 4 days) and colony counting.

For knockout generation, HEK293T cells were plated at 30–50% confluence and transduced with lentivirus at a multiplicity of infection of ~0.3. Medium was replaced after 24 h, and puromycin (1μg/mL) selection was applied 48-72 h post-infection for 3-5 days until uninfected control cells were eliminated. Single-cell clones were obtained by limiting dilution and validated by PCR amplification and Sanger sequencing.

Flow cytometry

Wild-type (WT) and KO cells were maintained in DMEM supplemented with 10% FBS inside a stationary incubator (37 °C, 5% CO2) and seeded into 6-well plates one day before transfection. For transfection, 4 μg of a single plasmid or a mixture of plasmids was diluted in 200 μL Opti-MEM. Separately, 12 μg PEI was diluted in 200 μL Opti-MEM and then added to the DNA solution. Transfection was performed according to previously established protocols.

After an additional 48 h of incubation at 37 °C, cells were harvested and washed three times with PBS. In assays employing CD59 as the GPI-AP reporter, cells were stained with an ABflo® 647-conjugated anti-CD59 antibody (ABclonal, cat. no. A22589; 1:20 dilution) for 1 h in the dark. After washing three times with PBS, cells were resuspended in 100 μL PBS for FACS. Data were acquired on a Novocyte 2060R cytometer with fluorescence detected at 488/525 nm (GFP channel) and 645/660 nm (APC channel). Approximately 40,000 cells were analyzed per sample. The gating strategy is shown in **Fig. S5**. Briefly, cells (WT or mutants) were gated to select living and single cells. Within this population, GFP-positive cells expressing the TGP-tagged GPI-T subunit were identified based on GFP fluorescence, and APC fluorescence was subsequently measured to evaluate CD59 surface expression. FACS results were analyzed using FlowJo software (BD Life Sciences, v10.8.1). Data represent the mean ± SD from three independent biological replicates, and statistical analyses were performed using GraphPad Prism 8.0.1. The expression levels of all mutant constructs were similar to that of the wild-type protein, as confirmed by Western blot analysis (**Fig. S10**).

Western blot

For immunoblotting assays, the five subunits of the *C. auris* GPI-T complex were individually cloned into pCAG expression vectors, with a C-terminal FLAG tag fused to one designated subunit. HEK293F cells (3×10^6 cells/mL) were co-transfected with equimolar amounts of the plasmids using PEI and incubated for 48 h at 37 °C. Cells were lysed for 1 h at 4 °C in lysis buffer supplemented with 1% (w/v) DDM, 0.1% (w/v) CHS, and 1% (v/v) protease inhibitor cocktail, followed by centrifugation at 15,000 × g for 40 minutes. Clarified lysates were mixed with loading buffer, separated on a 10 % SDS–PAGE, and then transferred to NC membranes. The membranes were immunoblotted with an anti-FLAG antibody (Proteintech; Cat No. 66008-4-lg, 1:5,000) to detect the FLAG-tagged subunit and with an anti-β-actin antibody (Proteintech; Cat No. 66009-1-Ig, 1:10,000) as a loading control, followed by incubation with an HRP-conjugated anti-mouse IgG secondary antibody (Proteintech; Cat No. SA00001-1, 1:5,000). Immunoreactive signals were visualized by enhanced chemiluminescence and captured using a TANON 5200CE imaging system.

Cryo-EM sample preparation and data collection

For single-particle cryo-EM analysis, 3 µL of *C. auris* GPI-T complex (~5 mg/mL) was applied onto glow-discharged Quantifoil carbon grids (R1.2/1.3 Au, 300 mesh). Grids were rapidly frozen in liquid ethane using an FEI Vitrobot Mark IV at 4°C, with 100% humidity and a 3-second blotting interval. Cryo-EM data were acquired on a Titan Krios (FEI) operated at 300 kV, equipped with a Gatan K3 detector and a GIF Quantum energy filter. Automated image collection utilized the EPU software package (FEI). Micrographs were acquired in super-resolution counting mode at physical pixel of 1.07 Å, with defocus ranging from −1.1 to −3.0 µm. A total exposure of 1.3 seconds was divided into 32 sub-frames, accumulating a total dose of 50 $e^-/Å^2$.

Cryo-EM image processing

Motion correction of the movie stacks was performed using MotionCor2 (Zheng et al., 2017), and subsequent contrast transfer function (CTF) estimation was carried out in cryoSPARC v3 (Punjani et al., 2017). Micrographs with poor quality or bad CTF fit (>4 Å) were excluded, yielding 1,283 micrographs for further analysis. Following particle auto-picking and multiple rounds of 2D classification, a subset of 405,935 high-quality particles was selected for initial model reconstruction. The resulting subset was then processed in RELION 3.0 (Zivanov et al., 2018) to generate an ab initio model, which was used as the initial reference for 3D classification. The class with high resolution features (170,112 particles) was selected for subsequent 3D refinement, particle polishing, and post-processing. Finally, a map with an overall resolution of 3.2 Å was obtained. The overall resolution was estimated using the gold-standard Fourier shell correlation (FSC) 0.143 criterion (Rosenthal and Henderson, 2003). The local resolution was estimated with ResMap (Kucukelbir et al., 2014). The corresponding workflow is provided in **Fig. S3B-F**.

Model building

Initial models were generated by rigid-body fitting of AlphaFold3-predicted structures in ChimeraX (Pettersen et al., 2021), followed by manual rebuilding in Coot (Emsley et al., 2010) guided by characteristic densities (bulky side chains, glycosylation sites, and disulfide bonds). Iterative real-space refinement was performed in PHENIX (Adams et al., 2010) with geometry restraints and secondary structure constraints. MOLPROBITY (Chen et al., 2010) was used to assess the final model. All structural figures were prepared using PyMOL (Schrödinger, LLC) and ChimeraX. Statistics of the 3D reconstructions and model refinements are shown in **Table. S1**.

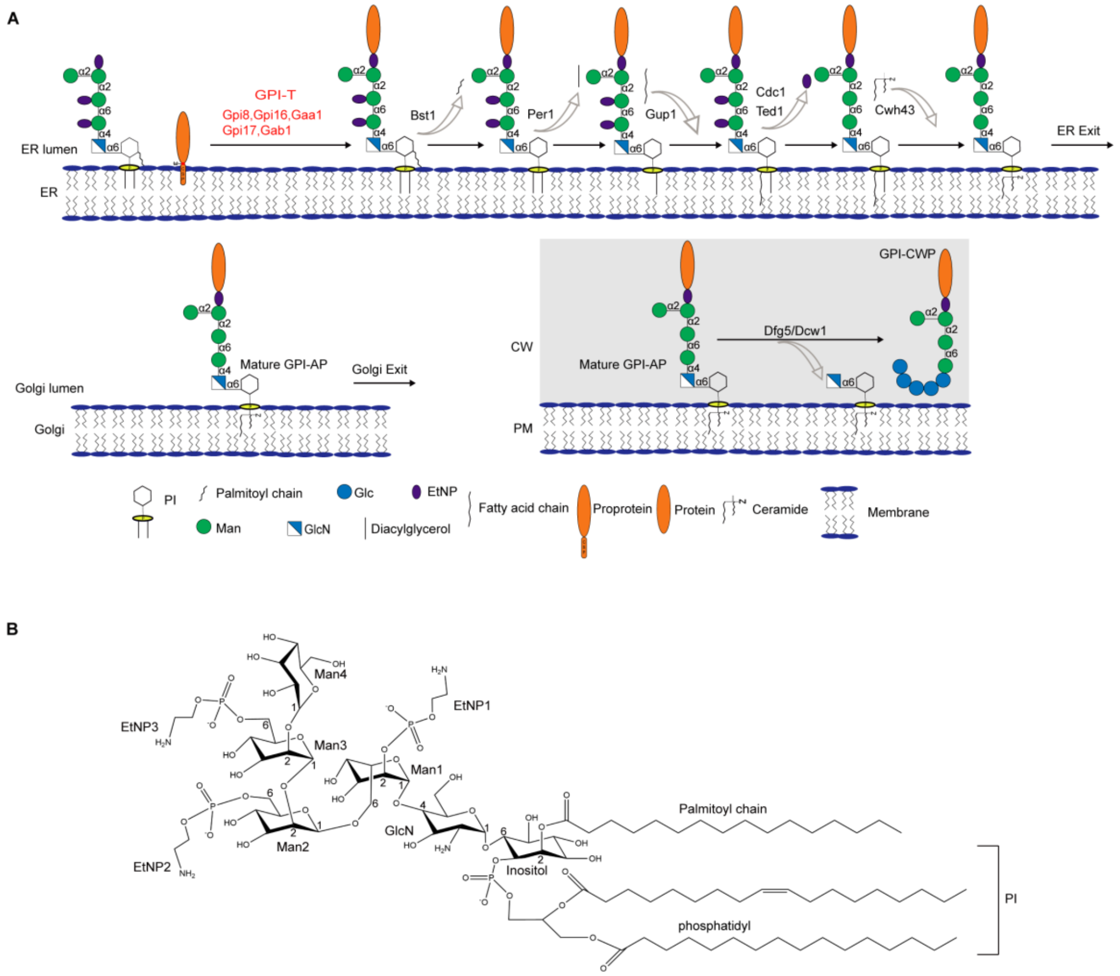

Figure S1. GPI-T in fungal GPI biosynthesis and chemical structure of GPI.

A, Schematic of fungal GPI biosynthesis. The GPI-T complex cleaves proproteins at the ω-site and catalyzes the replacement of the CSP with a preassembled GPI anchor within the endoplasmic reticulum (ER) lumen. Subsequently, the newly formed GPI-AP undergoes sequential lipid and glycan remodeling steps in the ER, including inositol deacylation, fatty-acid remodeling, and mannosyl side-chain glycan remodeling. In the Golgi, additional maturation reactions further modify both the glycan core and lipid moiety, generating transport-competent GPI-APs. Mature GPI-anchored proteins are then directed to the plasma membrane or incorporated into the fungal cell wall (GPI-CWPs). Glycosidic linkages within the core GPI-anchor glycan are annotated.

B, The GPI core in fungi contains a phosphatidylinositol (PI), a palmitoyl chain, a glucosamine (GlcN), three mannoses (Man1-3), each modified by an ethanolamine phosphate (EtNP1-3), and an additional fourth mannose (Man4).

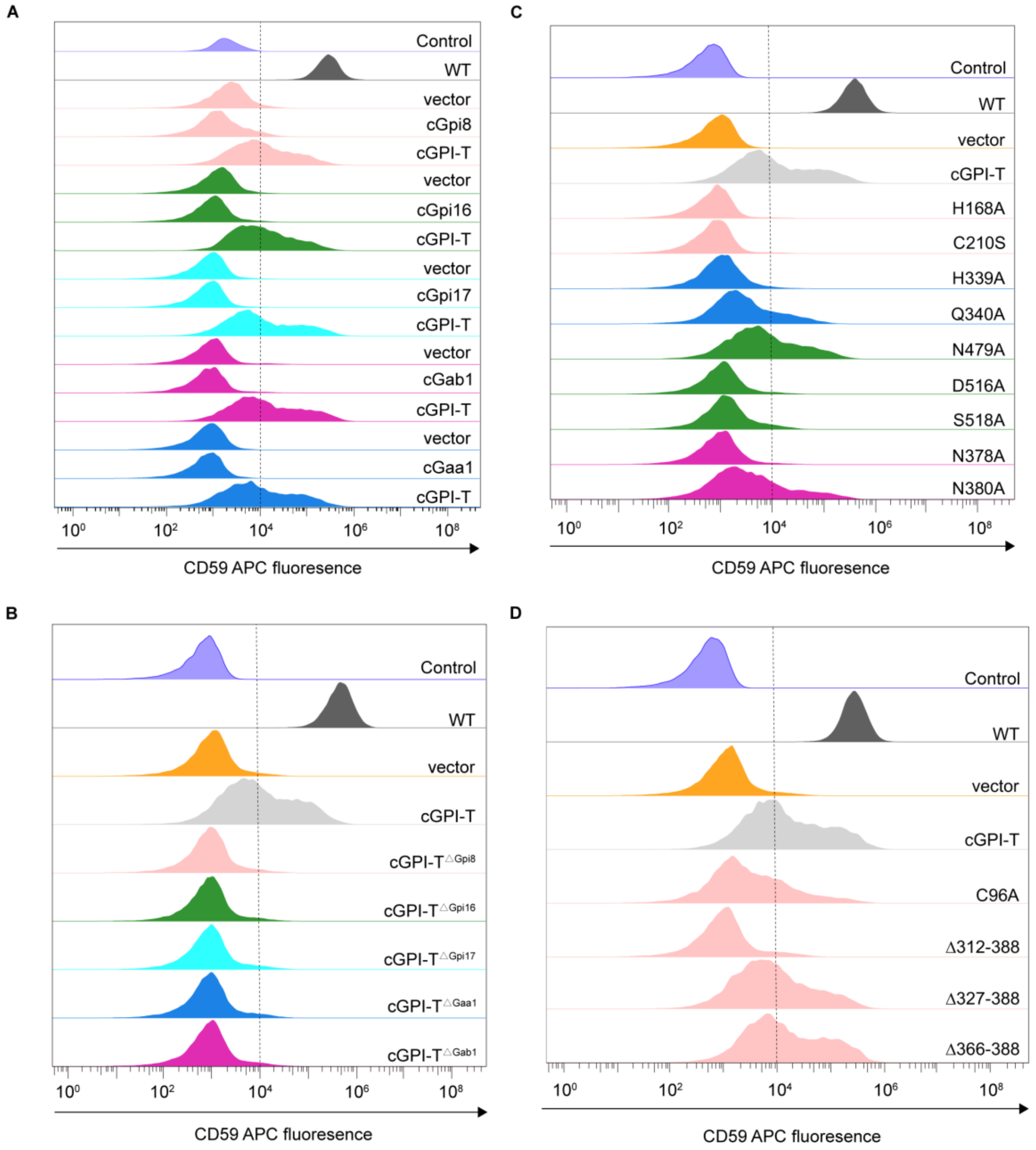

Figure S2. Representative FACS results for the in vivo functional analysis of *C. auris* GPI-T.

A-D, In vivo functional analysis of GPI-T and GPI-T mutations relative to the WT and control, with representative FACS results from three independent replicates shown. "Control" (purple) represents the background fluorescence from unstained HEK293^{WT} cells. "WT" (black) indicates CD59 staining in HEK293^{WT} cells. "Vector" corresponds to CD59 staining in KO cells transfected with the Thermostable Green Protein (TGP)-pCAG empty vector.

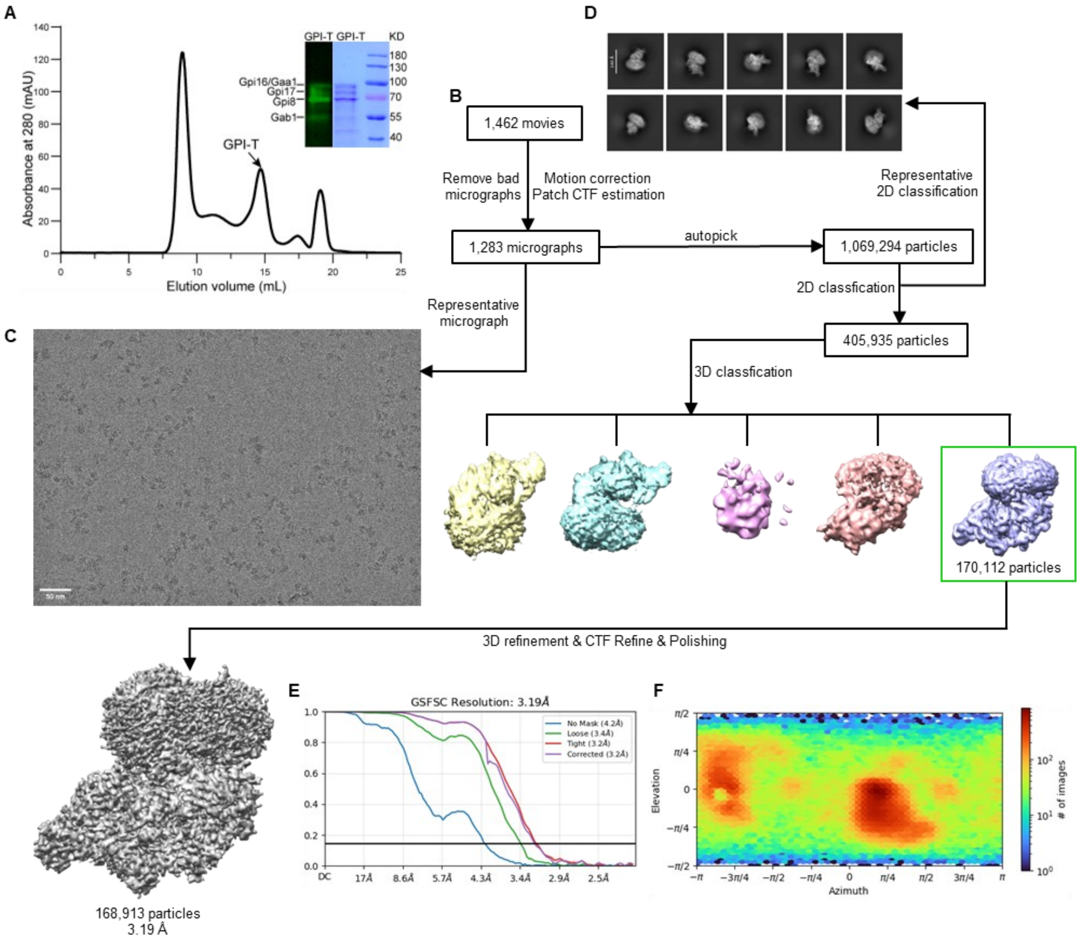

Figure S3. Cryo-EM analysis of *C. auris* GPIT.

A, SDS-PAGE analysis of the GPI-T fraction from the elution profile, visualized by in-gel fluorescence (left) and Coomassie staining (right). This fraction contains all five subunits.

B, Flowchart showing the steps of cryo-EM data acquisition and processing for *C. auris* GPI-T. See **Materials and Methods** for details.

C, A representative cryo-EM micrograph of GPI-T.

D, Representative 2D class averages.

E, Gold-standard Fourier shell correlation (FSC) curve showing the 3D refinement of GPI-T.

F, Angular distribution of particles used in the final 3D reconstruction.

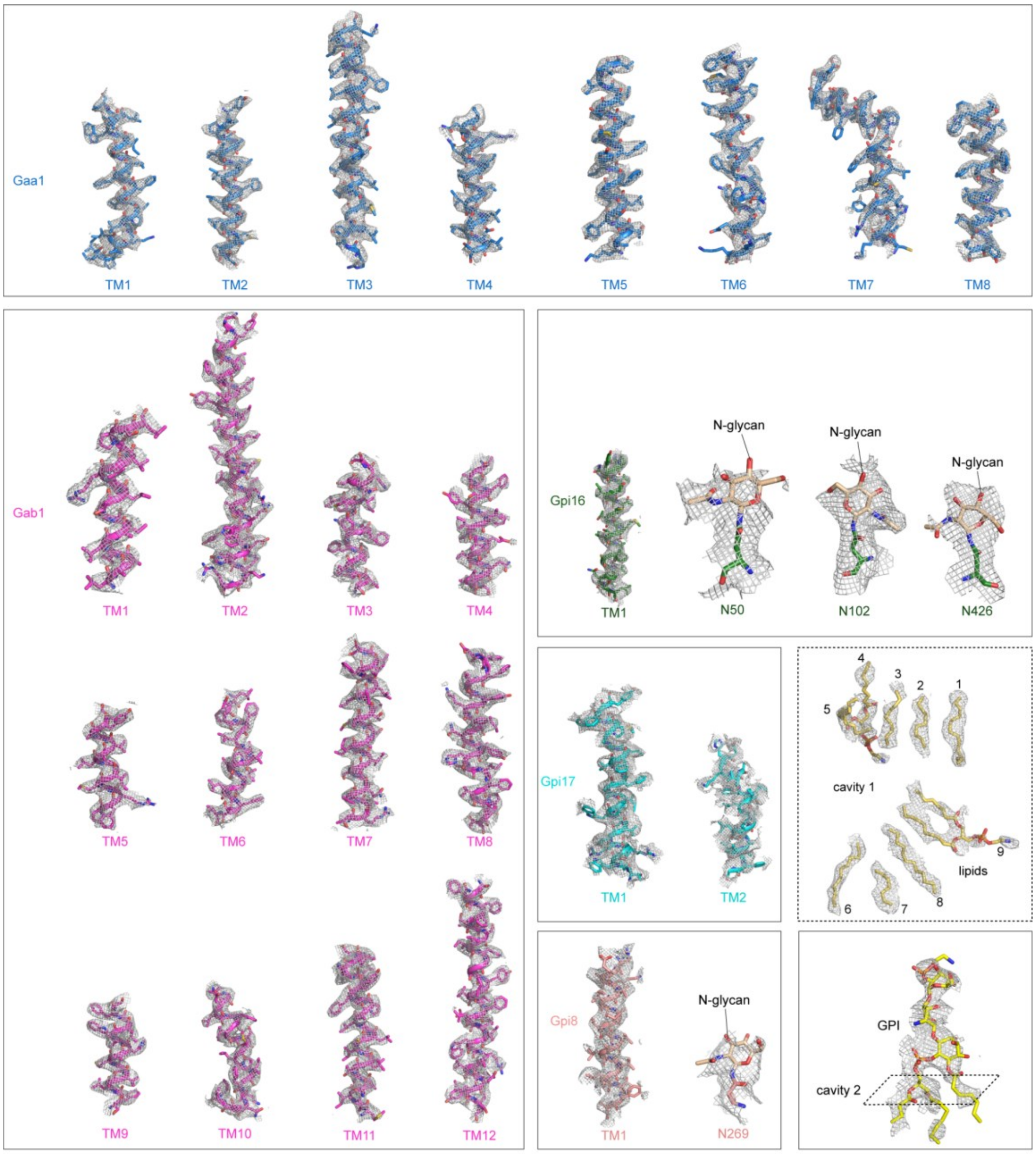

Figure S4. Density fitting of *C. auris* GPI-T model in example regions.

Cryo-EM density of representative protein regions contains TM1-8 of Gaa1, TM1-12 of Gab1, TM1-2 of Gpi17, TM1 and three N-glycosylation sites of Gpi16, TM1 and one N-glycosylation site of Gpi8, lipids in cavity 1, and a GPI ligand extending from cavity 2 to the luminal domain.

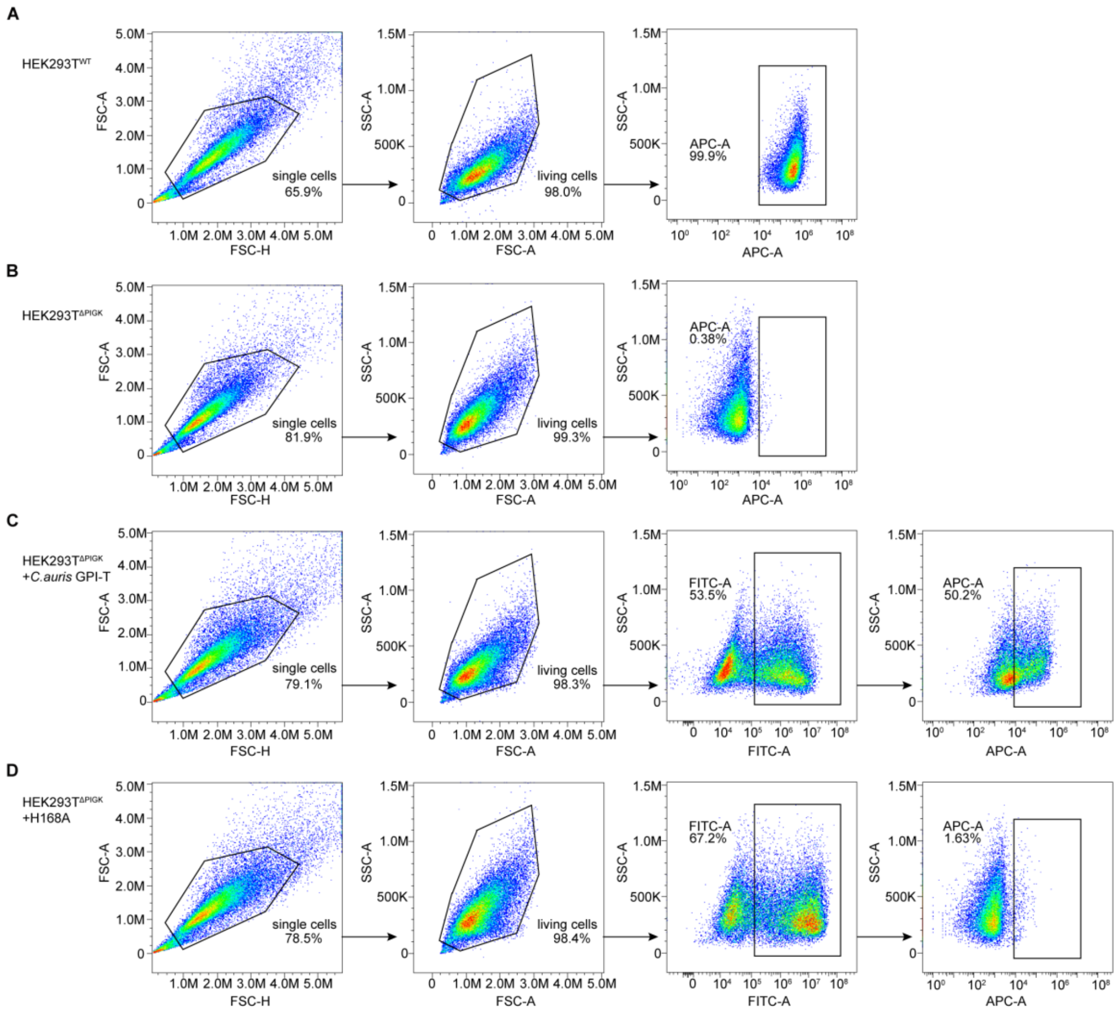

Figure S5. Representative gating strategies in FACS.

A-B, For the control, single and living cells were isolated by gating, and the population was then analyzed for APC fluorescence emission (645/660 nm), which indicated antibody-mediated CD59 surface staining.

C-D, For the experimental group, single and living cells were first gated, followed by GFP fluorescence gating to isolate cells expressing TGP-tagged subunits (*C. auris* GPI-T WT or mutants). The GFP-positive population was then analyzed for APC positivity, indicating CD59 surface stainin. FSC-A/H: forward scatter area/height; SSC-A: side scatter area; FITC: fluorescein isothiocyanate (channel for GFP detection); APC: allophycocyanin (channel for CD59 detection).

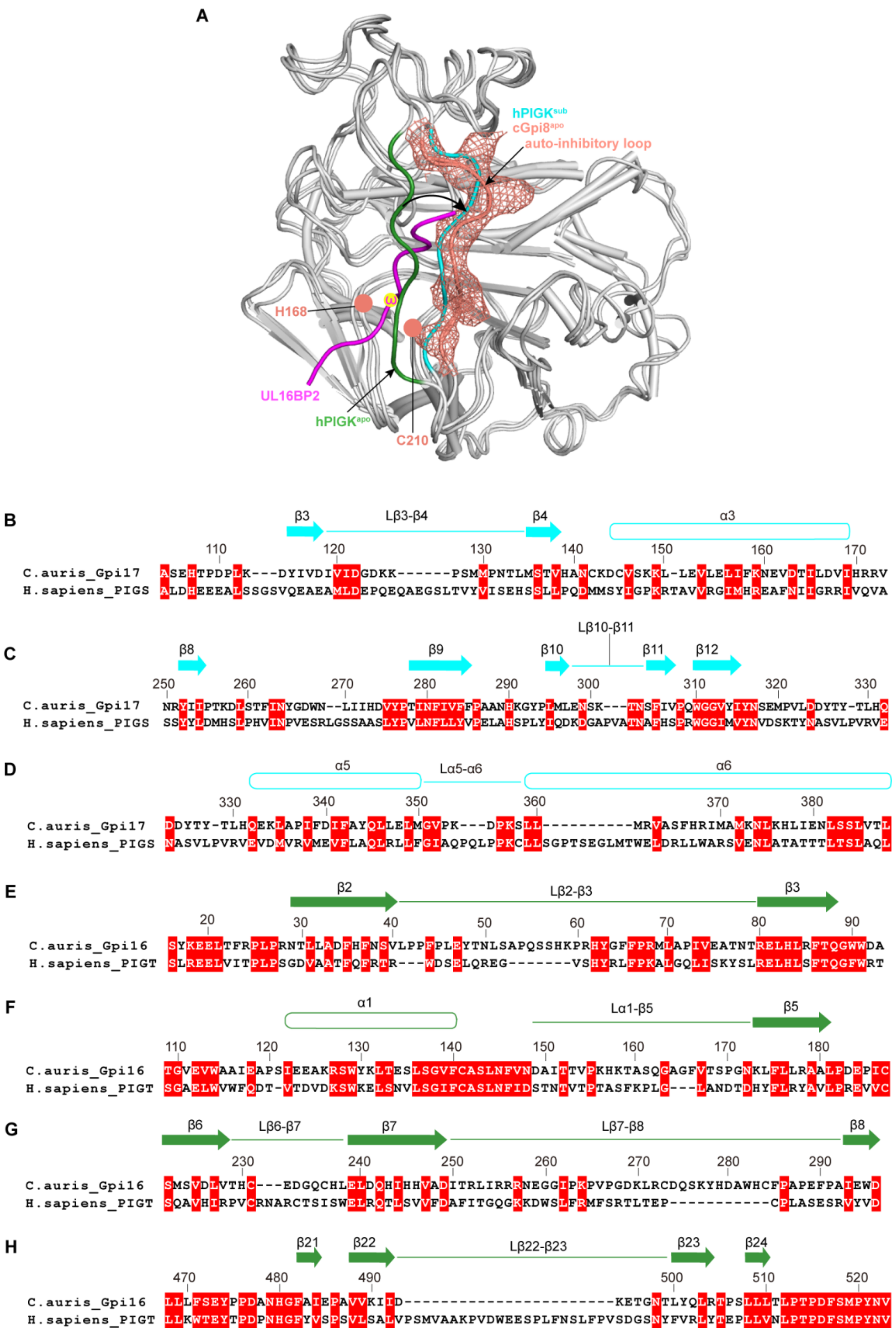

Figure S6. Analysis of the auto-inhibitory loop and sequence comparison of divergent regions between *C. auris* and human GPI-T.

A, Comparison of the auto-inhibitory loop between *C. auris* GPI-T and the two conformations of the human counterpart. The structure of human UL16BP2 (magenta)

is superimposed onto the apo form of *C. auris* Gpi8 (cGpi8^{apo}, salmon). The apo hPIGK (hPIGK^{apo}, green, PDB:7WLD) shows the inhibitory loop obstructing substrate access, while the substrate-bound form (hPIGK^{sub}, light blue, PDB:8IMY) adopts an active conformation. The captured cGpi8^{apo} adopts a conformation similar to the active state. The catalytic dyad (H168 and C210, salmon spheres) and the ω-site of UL16BP2 (red) are indicated.

B-H, Sequence comparison of divergent regions in the inter-subunit interfaces between the *C. auris* and human GPI-T (**Fig. 2E-H**). Corresponding structural elements are annotated in the sequence alignments.

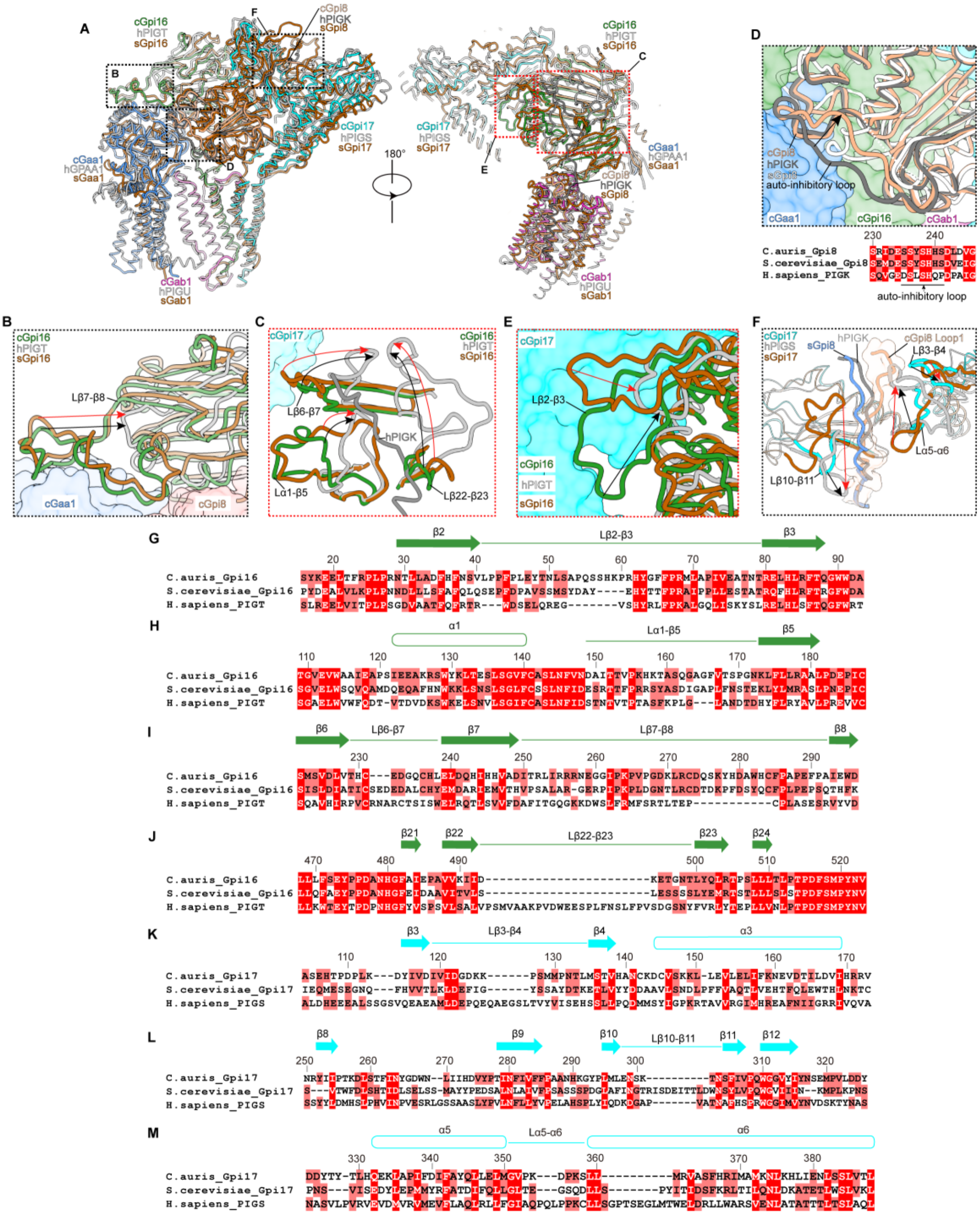

Figure S7. Structural and sequence comparisons of GPI-T from *C. auris*, *S. cerevisiae*, and humans.

A, Overall structural comparison from *C. auris, S. cerevisiae*, and human GPI-T.

B–C, Close-up view of the divergent regions highlighted in **A**, showing structural differences at the Gpi16–Gaa1–Gpi8 (**B**) and Gpi8–Gpi16 (**C**) interfaces. Structural differences relative to human GPI-T are indicated by black arrows (*C. auris*) and red arrows (*S. cerevisiae*).

D, Structural and sequence comparisons of the Gpi8 auto-inhibitory loop in *C. auris*, *S. cerevisiae*, and humans. The white dashed line denotes the unresolved auto-inhibitory loop in *S. cerevisiae*. A major structural difference is highlighted by an

arrow.

E–**F,** Close-up view of the structural differences at the Gpi16–Gpi17 (**E**) and Gpi8–Gpi17 (**F**) interfaces. Structural differences from human GPI-T are marked by black (*C. auris*) and red (*S. cerevisiae*) arrows.

G–**M,** Sequence alignments of the regions shown in panels **B**–**C** and **E**–**F** from *C. auris, S. cerevisiae*, and human GPI-T. The corresponding secondary structure elements are annotated in the alignments.

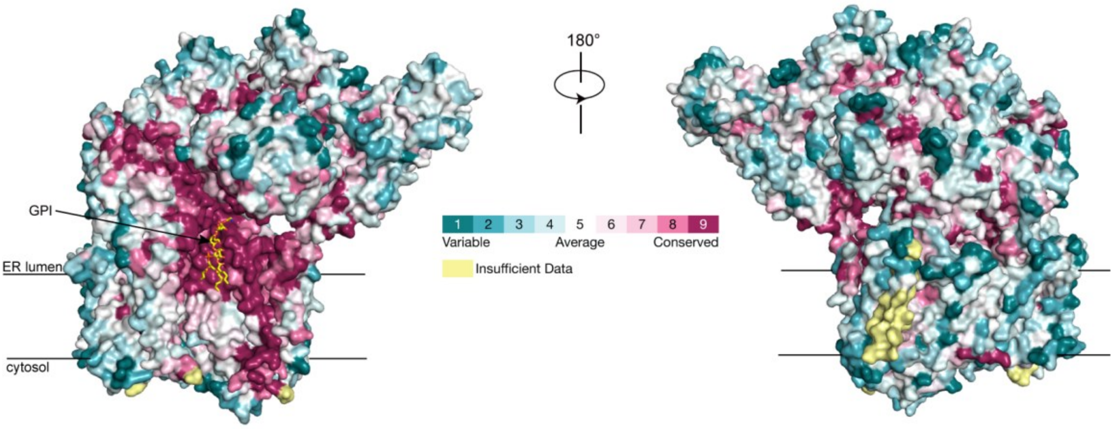

Figure S8. Sequence conservation of GPI-T illustrated on *C. auris* GPI-T structure. Surface representation of sequence conservation on the GPI-T structure, shown in two orientations (front and back) and colored by conservation level, predicted by the ConSurf server.

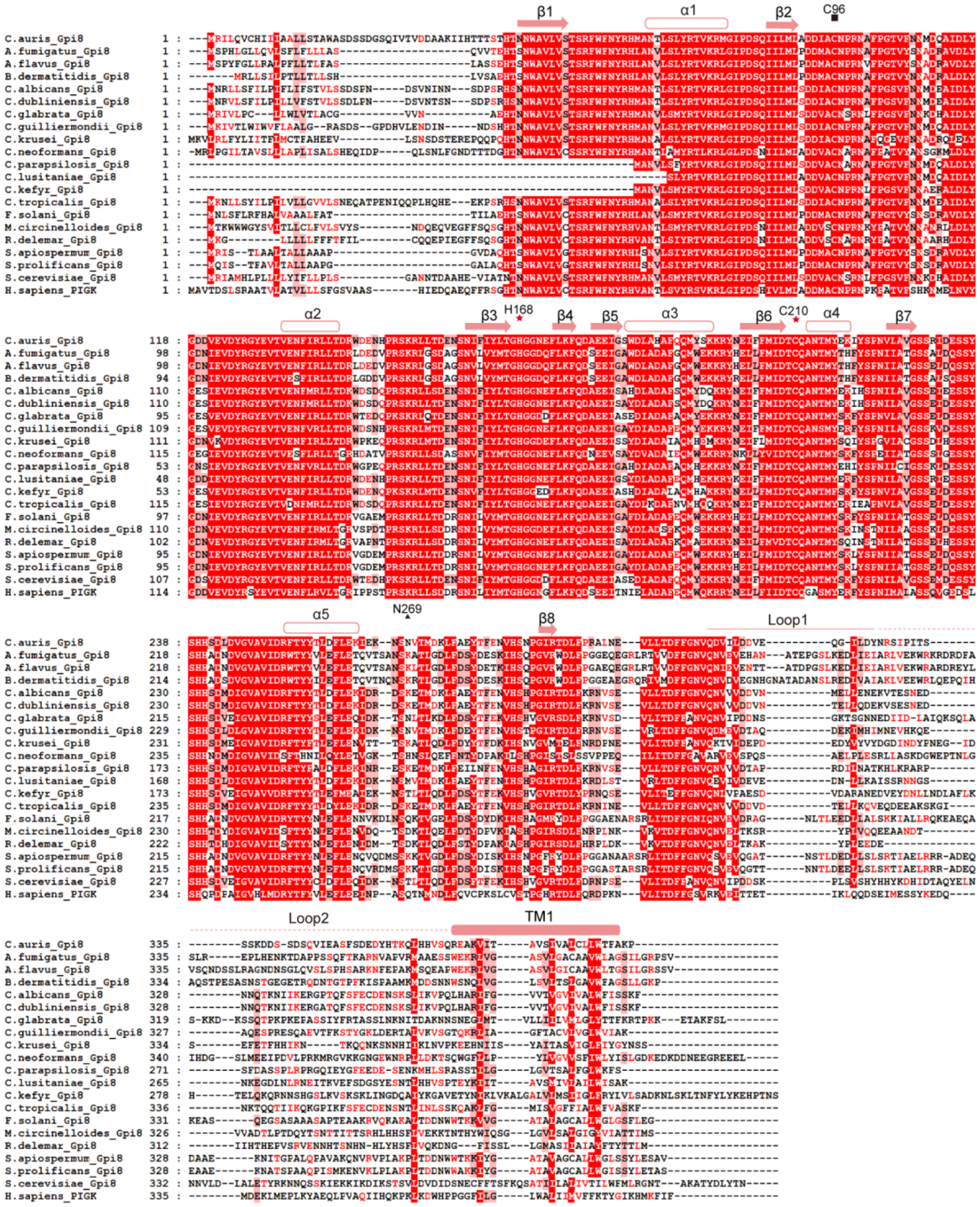
β1
α1
β2
C96
α2
β3
H168
β4
β5
α3
β6
C210
α4
β7
α5
N269
β8
Loop1
Loop2
TM1
C.auris_Gpi8
A.fumigatus_Gpi8
A.flavus_Gpi8
B.dermatitidis_Gpi8
C.albicans_Gpi8
C.dubliniensis_Gpi8
C.glabrata_Gpi8
C.guilliermondii_Gpi8
C.krusei_Gpi8
C.neoformans_Gpi8
C.parapsilosis_Gpi8
C.lusitaniae_Gpi8
C.kefyr_Gpi8
C.tropicalis_Gpi8
F.solani_Gpi8
M.circinelloides_Gpi8
R.delemar_Gpi8
S.apiospermum_Gpi8
S.prolificans_Gpi8
S.cerevisiae_Gpi8
H.sapiens_PIGK

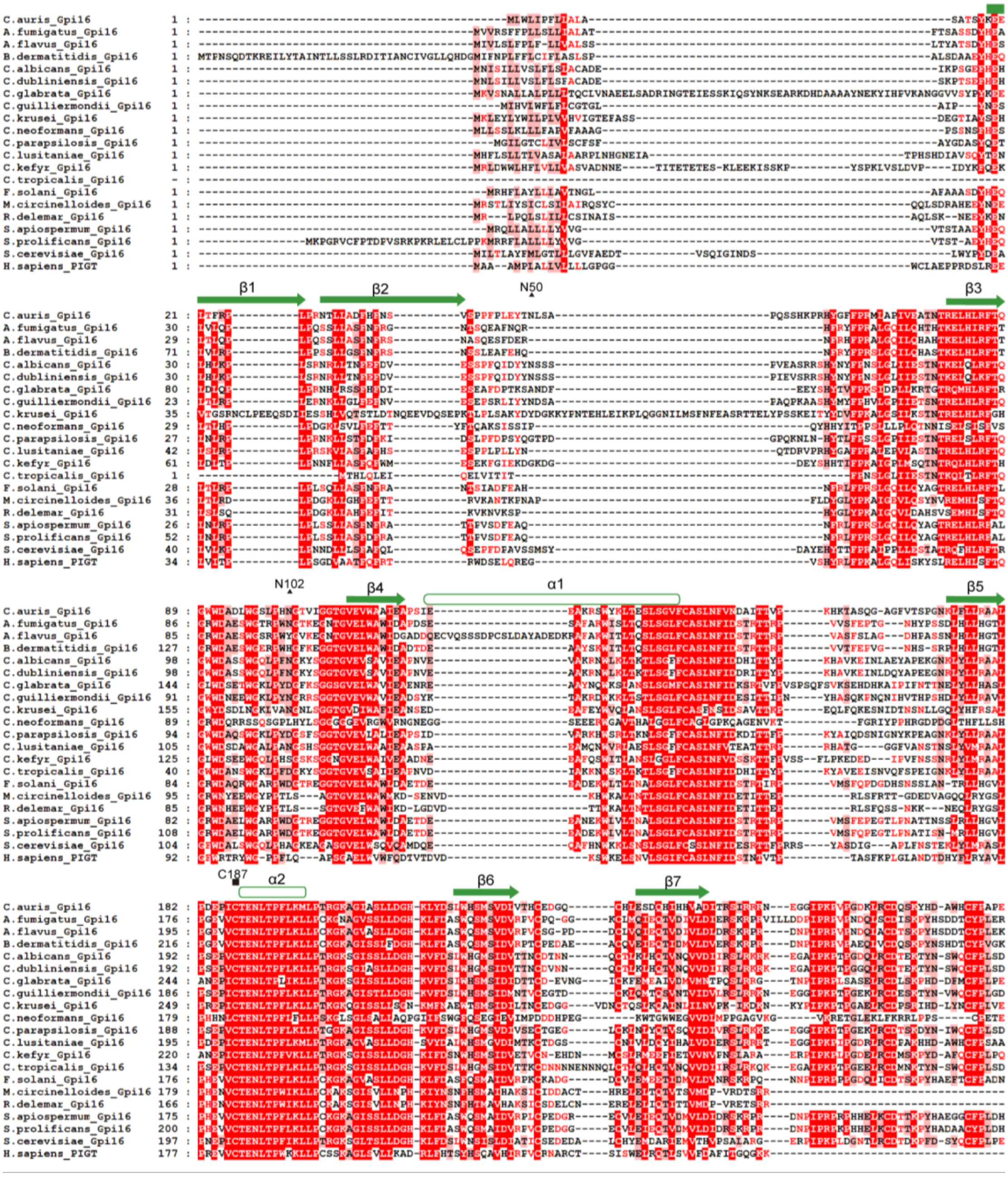
C.auris_Gpi16
A.fumigatus_Gpi16
A.flavus_Gpi16
B.dermatitidis_Gpi16
C.albicans_Gpi16
C.dubliniensis_Gpi16
C.glabrata_Gpi16
C.guilliermondii_Gpi16
C.krusei_Gpi16
C.neoformans_Gpi16
C.parapsilosis_Gpi16
C.lusitaniae_Gpi16
C.kefyr_Gpi16
C.tropicalis_Gpi16
F.solani_Gpi16
M.circinelloides_Gpi16
R.delemar_Gpi16
S.apiospermum_Gpi16
S.prolificans_Gpi16
S.cerevisiae_Gpi16
H.sapiens_PIGT
β1
β2
N50
β3
N102
β4
α1
β5
C187
α2
β6
β7

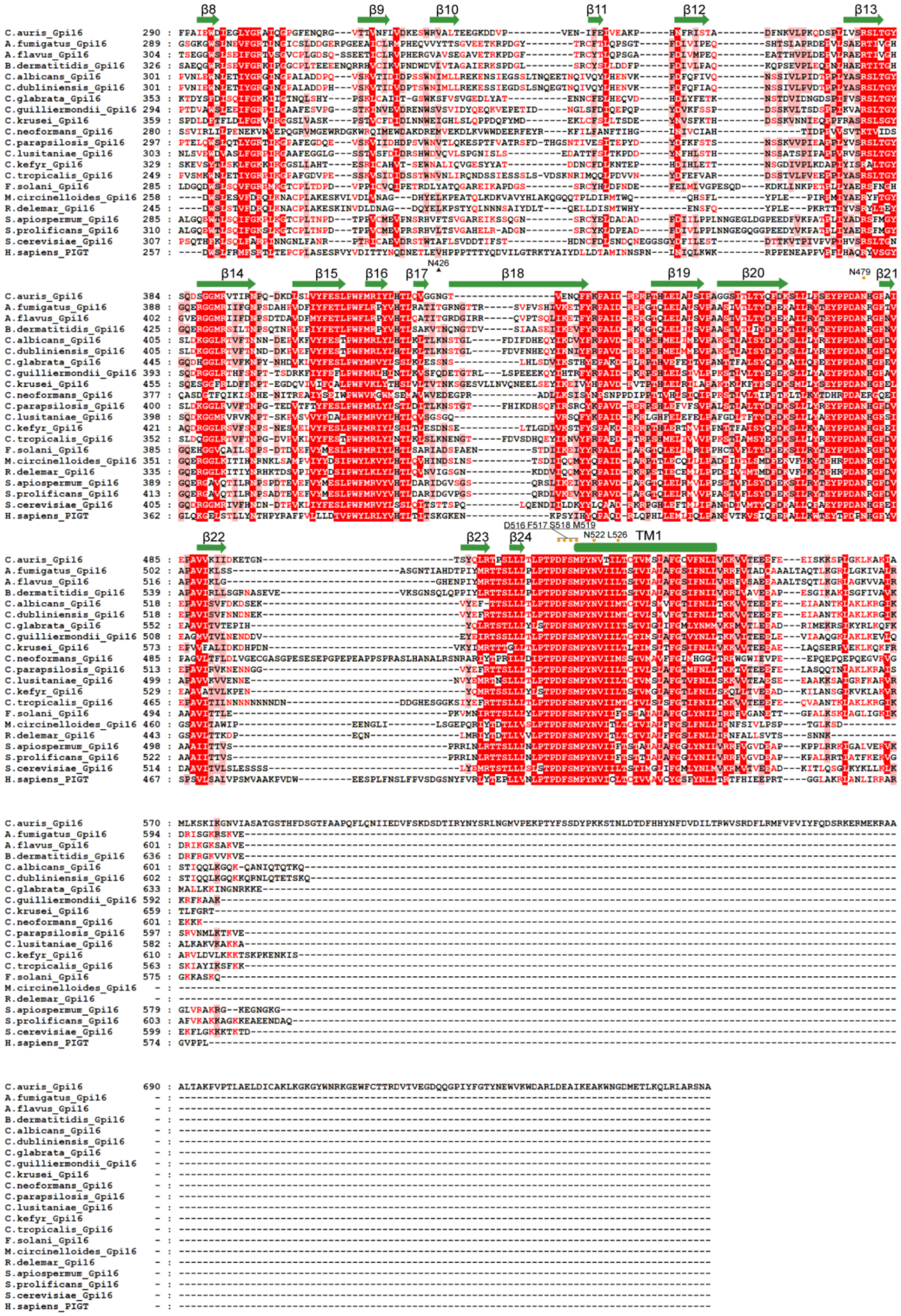
β8
β9
β10
β11
β12
β13
β14
β15
β16
β17
N426
β18
β19
β20
N479
β21
β22
D516 F517 S518 M519
β23
β24
N522 L526
TM1

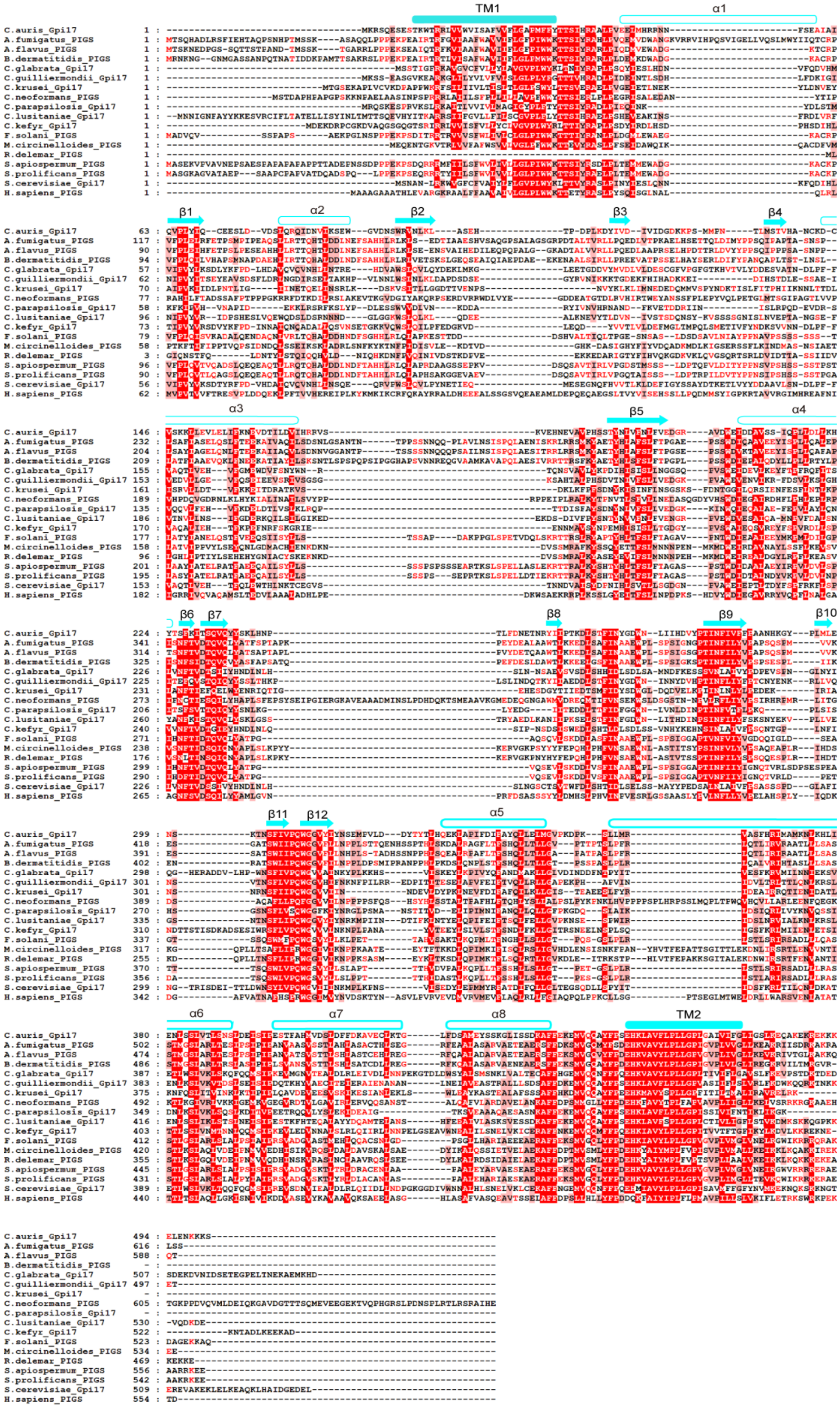
TM1
α1
β1
α2
β2
β3
β4
α3
β5
α4
β6
β7
β8
β9
β10
β11
β12
α5
α6
α7
α8
TM2
C.auris_Gpi17
A.fumigatus_PIGS
A.flavus_PIGS
B.dermatitidis_PIGS
C.glabrata_Gpi17
C.guilliermondii_Gpi17
C.krusei_Gpi17
C.neoformans_PIGS
C.parapsilosis_Gpi17
C.lusitaniae_Gpi17
C.kefyr_Gpi17
F.solani_PIGS
M.circinelloides_PIGS
R.delemar_PIGS
S.apiospermum_PIGS
S.prolificans_PIGS
S.cerevisiae_Gpi17
H.sapiens_PIGS

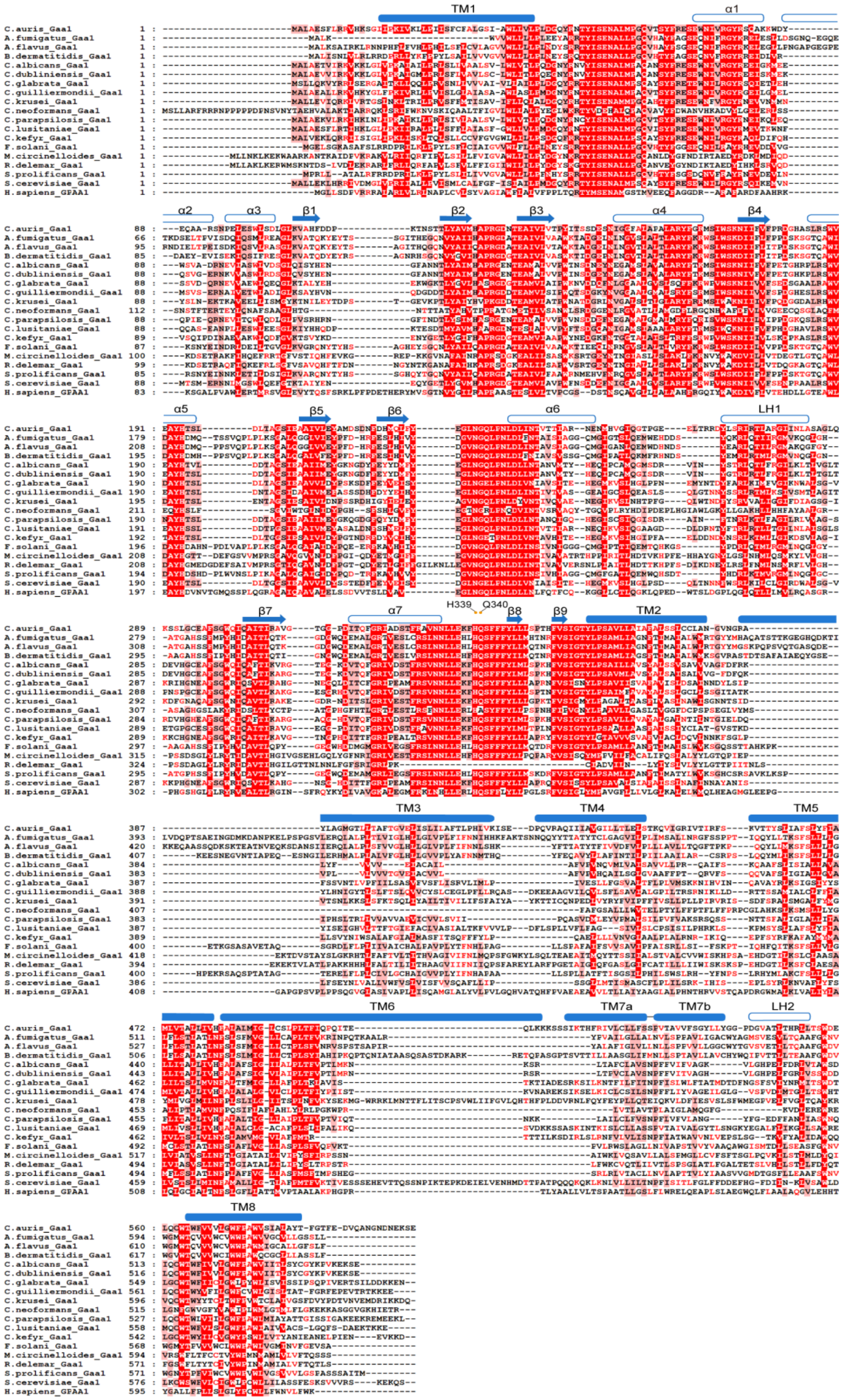

TM1
α1
α2
α3
β1
β2
β3
α4
β4
α5
β5
β6
α6
LH1
β7
α7
H339
Q340
β8
β9
TM2
TM3
TM4
TM5
TM6
TM7a
TM7b
LH2
TM8
C.auris_Gaa1
A.fumigatus_Gaa1
A.flavus_Gaa1
B.dermatitidis_Gaa1
C.albicans_Gaa1
C.dubliniensis_Gaa1
C.glabrata_Gaa1
C.guilliermondii_Gaa1
C.krusei_Gaa1
C.neoformans_Gaa1
C.parapsilosis_Gaa1
C.lusitaniae_Gaa1
C.kefyr_Gaa1
F.solani_Gaa1
M.circinelloides_Gaa1
R.delemar_Gaa1
S.prolificans_Gaa1
S.cerevisiae_Gaa1
H.sapiens_GPAA1

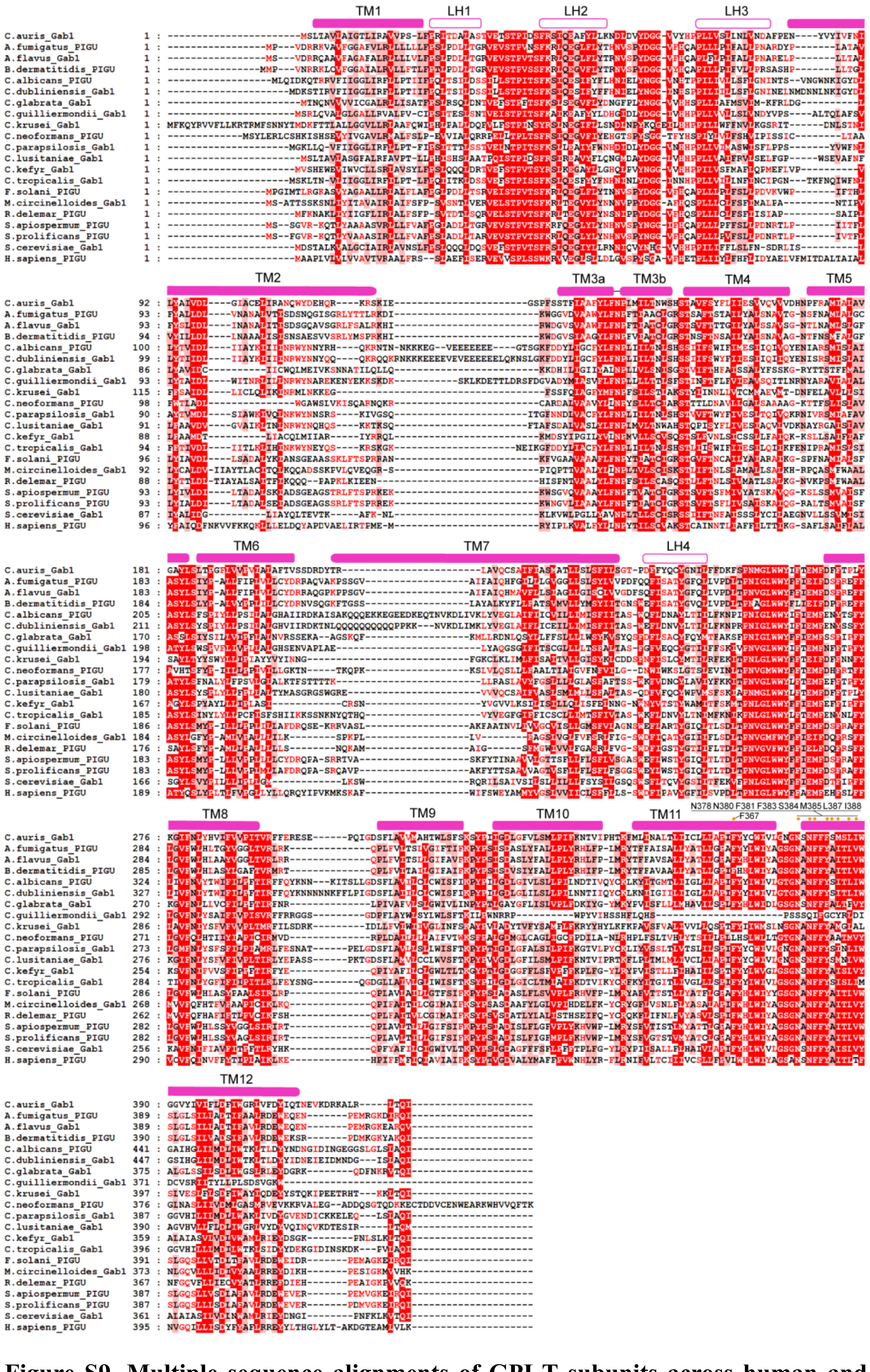

Figure S9. Multiple sequence alignments of GPI-T subunits across human and fungal species.

The primary sequence alignment was generated using Clustalx and visualized with GeneDoc. Functional residues are highlighted as follows: red stars, catalytic dyad in Gpi8; orange circles, GPI binding site; black triangles, glycosylation sites; black rectangles, the two cysteine residues forming the intermolecular disulfide bond between Gpi8 and Gpi16. Secondary structural elements, derived from the *C. auris* GPIT structure, are shown above the sequences. Protein sequences are sourced from *Homo sapiens* and multiple fungal species, including *C. auris*, *A. fumigatus*, *A. flavus*, *B. dermatitidis*, *C. albicans*, *C. dubliniensis*, *C. glabrata*, *C. guilliermondii*, *C. krusei*, *C. neoformans*, *C. parapsilosis*, *C. lusitaniae*, *C. kefyr*, *C. tropicalis*, *F. solani*, *M. circinelloides*, *R. delemar*, *S. apiospermum*, *S. cerevisiae*, *and S. prolificans*.

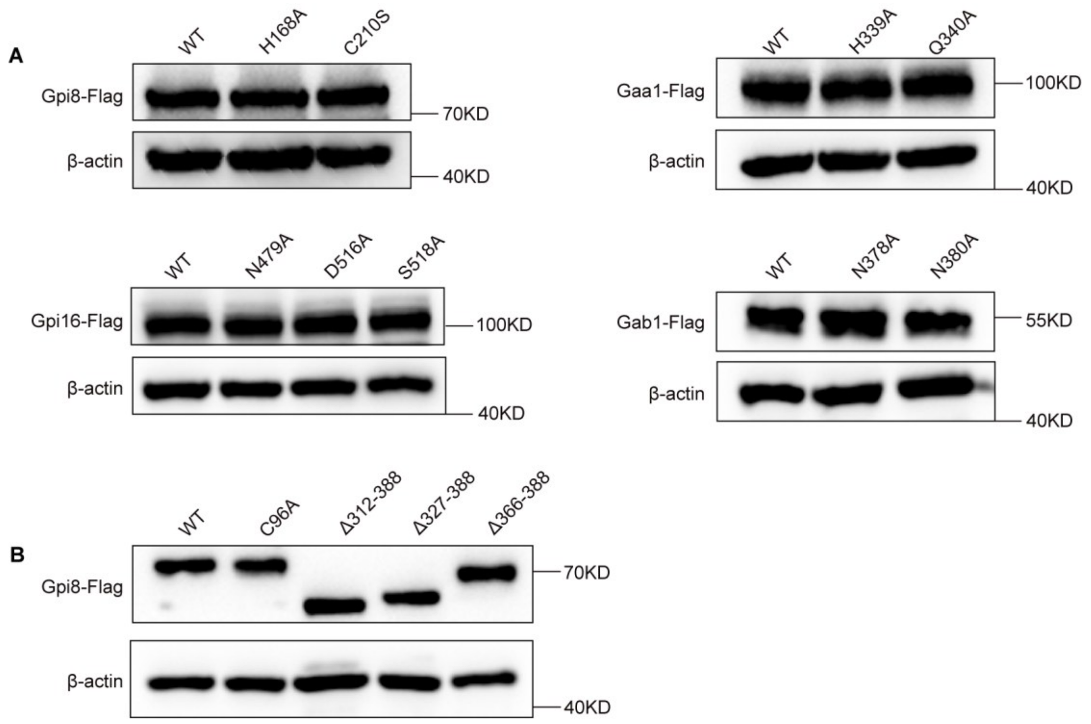

Figure S10. Immunoblot analysis showing the expression of *C. auris* GPI-T and its mutants.

A, GPI-binding site mutants corresponding to **Fig. 1K**.

B, Gpi8 C-terminal truncations and a point mutant corresponding to **Fig. 2K**.

Table S1. Cryo-EM data collection, refinement and validation statistics.

	Candida auris GPI-T (EMDB-66656) (PDB 9X8O)
**Data collection and processing**	
Magnification	130,000
Voltage (kV)	300
Electron exposure (e–/Å²)	50
Defocus range (μm)	-1.1 to -3
Pixel size (Å)	1.07
Symmetry imposed	C1
Initial particle images (no.)	1,069,294
Final particle images (no.)	168,913
Map resolution (Å)	3.19
FSC threshold	0.143
**Refinement**	
Initial model used (PDB code)	AlphaFold3
Model resolution (Å)	3.3
FSC threshold	0.5
Map sharpening B factor (Å²)	98.5
Model composition	
Non-hydrogen atoms	18693
Protein residues	2315
Ligands	1 GPI<br>4 N-glycans<br>15 Lipids
B factor (Å²)	
Protein	52.69
Ligand	77.00
R.m.s. deviations	
Bond lengths (Å)	0.003
Bond angles (°)	0.527
Validation	
MolProbity score	1.72
Clash score	6.73
Poor rotamers (%)	0.00
Ramachandran plot	
Favored (%)	95.00
Allowed (%)	5.00
Outliers (%)	0.00

References

Adams, P.D., Afonine, P.V., Bunkóczi, G., *et al.* (2010). PHENIX: a comprehensive Python-based system for macromolecular structure solution. Acta crystallographica Section D, Biological crystallography. 66, 213-221.

Cai, H., Yao, H., Li, T., *et al.* (2020). An improved fluorescent tag and its nanobodies for membrane protein expression, stability assay, and purification. Communications biology. 3, 753.

Chen, V.B., Arendall, W.B., 3rd, Headd, J.J., *et al.* (2010). MolProbity: all-atom structure validation for macromolecular crystallography. Acta crystallographica Section D, Biological crystallography. 66, 12-21.

Close, D.W., Paul, C.D., Langan, P.S., *et al.* (2015). Thermal green protein, an extremely stable, nonaggregating fluorescent protein created by structure-guided surface engineering. Proteins. 83, 1225-1237.

Emsley, P., Lohkamp, B., Scott, W.G., *et al.* (2010). Features and development of Coot. Acta crystallographica Section D, Biological crystallography. 66, 486-501.

Kucukelbir, A., Sigworth, F.J., and Tagare, H.D. (2014). Quantifying the local resolution of cryo-EM density maps. Nature methods. 11, 63-65.

Pettersen, E.F., Goddard, T.D., Huang, C.C., *et al.* (2021). UCSF ChimeraX: Structure visualization for researchers, educators, and developers. Protein science : a publication of the Protein Society. 30, 70-82.

Punjani, A., Rubinstein, J.L., Fleet, D.J., *et al.* (2017). cryoSPARC: algorithms for rapid unsupervised cryo-EM structure determination. Nature methods. 14, 290-296.

Rosenthal, P.B., and Henderson, R. (2003). Optimal determination of particle orientation, absolute hand, and contrast loss in single-particle electron cryomicroscopy. Journal of molecular biology. 333, 721-745.

Sanjana, N.E., Shalem, O., and Zhang, F. (2014). Improved vectors and genome-wide libraries for CRISPR screening. Nature methods. 11, 783-784.

Shalem, O., Sanjana, N.E., Hartenian, E., *et al.* (2014). Genome-scale CRISPR-Cas9 knockout screening in human cells. Science (New York, NY). 343, 84-87.

Wang, T., Wei, J.J., Sabatini, D.M., *et al.* (2014). Genetic screens in human cells using the CRISPR-Cas9 system. Science (New York, NY). 343, 80-84.

Zheng, S.Q., Palovcak, E., Armache, J.P., *et al.* (2017). MotionCor2: anisotropic correction of beam-induced motion for improved cryo-electron microscopy. Nature methods. 14, 331-332.

Zivanov, J., Nakane, T., Forsberg, B.O., *et al.* (2018). New tools for automated high-resolution cryo-EM structure determination in RELION-3. eLife. 7.
